# Polyfunctional Type-1, -2, and -17 CD8^+^ T Cell Responses to Apoptotic Self-Antigens Correlate with the Chronic Evolution of Hepatitis C Virus Infection

**DOI:** 10.1371/journal.ppat.1002759

**Published:** 2012-06-21

**Authors:** Debora Franceschini, Paola Del Porto, Silvia Piconese, Emanuele Trella, Daniele Accapezzato, Marino Paroli, Stefania Morrone, Enza Piccolella, Enea Spada, Alfonso Mele, John Sidney, Alessandro Sette, Vincenzo Barnaba

**Affiliations:** 1 Dipartimento di Medicina Interna e Specialità Mediche, Sapienza Università di Roma, Rome, Italy; 2 Dipartimento di Biologia e Biotecnologie “Charles Darwin”, Sapienza Università di Roma, Rome, Italy; 3 Dipartimento di Scienze e Biotecnologie Medico-Chirurgiche, Sapienza Università di Roma, Rome, Italy; 4 Dipartimento di Medicina Sperimentale, Sapienza Università di Roma, Rome, Italy; 5 National Centre of Epidemiology, Surveillance and Health Promotion, Istituto Superiore di Sanità, Rome, Italy; 6 La Jolla Institute for Allergy and Immunology, San Diego, California, United States of America; 7 Istituto Pasteur - Fondazione Cenci Bolognetti, Rome, Italy; 8 Fondazione Andrea Cesalpino, Rome, Italy; Nationwide Children's Hospital, United States of America

## Abstract

Caspase-dependent cleavage of antigens associated with apoptotic cells plays a prominent role in the generation of CD8^+^ T cell responses in various infectious diseases. We found that the emergence of a large population of autoreactive CD8^+^ T effector cells specific for apoptotic T cell-associated self-epitopes exceeds the antiviral responses in patients with acute hepatitis C virus infection. Importantly, they endow mixed polyfunctional type-1, type-2 and type-17 responses and correlate with the chronic progression of infection. This evolution is related to the selection of autoreactive CD8^+^ T cells with higher T cell receptor avidity, whereas those with lower avidity undergo prompt contraction in patients who clear infection. These findings demonstrate a previously undescribed strict link between the emergence of high frequencies of mixed autoreactive CD8^+^ T cells producing a broad array of cytokines (IFN-γ, IL-17, IL-4, IL-2…) and the progression toward chronic disease in a human model of acute infection.

## Author Summary

The emergence of a large population of mixed polyfunctional (type-1, -2, -17) CD8^+^ T cell effector responses specific for apoptotic T cell-associated self-epitopes rather than the dysfunction or altered quality of virus-specific CD8^+^ T cells is associated with the progression toward chronic disease in the human model of acute HCV infection. The chronic evolution is associated with the selection of autoreactive CD8^+^ T cells with higher T cell receptor avidity, whereas those with lower avidity undergo prompt contraction, as seen in patients undergoing infection resolution. We suggest that these autoreactive responses are secondary to the viral persistence and can participate to the HCV-related immunopathology. This data has implications for the prognosis and therapy of infections undergoing chronic evolution.

## Introduction

The fate of the enormous number of apoptotic cells that derive from effector Tcells undergoing apoptosis after performing their functions during acute or chronic infections remain to be determined [1], [2]. Phagocytosis of apoptotic cells by dendritic cells (DCs) leads to the processing of apoptotic cell-associated antigens and the cross-presentation of the resulting peptides on major histocompatibility complex (MHC) class I molecules [3]–[6]. This phenomenon seems crucial for inducing either cross-priming or cross-tolerance of CD8^+^T cells, based on the presence or absence of various infectious or danger signals influencing the switch from tolerogenic immature (i)DCs to mature (m)DCs with high stimulatory and migratory capacities [3]–[7]. In previous studies, we found that the proteome of apoptotic T cells includes prominent caspase-cleaved cellular proteins and that a high proportion of distinct epitopes in these fragments (apoptotic epitopes) can be cross-presented by DCs to a wide repertoire of autoreactive CD8^+^ T cells [8]. Recent reports have confirmed the role of caspase cleavage in the processing and presentation of epitopes that are derived from apoptotic cells in different models [9]–[11]. In chronic HIV infection, these autoreactive CD8^+^ T cells correlate with the proportion of apoptotic CD4^+^ T cells *in vivo* and are involved in establishing polyclonal T cell activation that in the long run results in generalized T cell dysfunction/depletion [8]. In addition, apoptotic cells derived from activated T cells (in contrast to those derived from resting T cells or from non-lymphoid cells) retain the expression of CD40 ligand (L) and can then condition CD40^+^ DCs to acquire high capacities to prime or cross-prime autoreactive T cells [12], [13]. This mechanism is consistent with the evidence that the signals provided by CD40L^+^ apoptotic cells and not those provided by conventional apoptotic cells facilitate the emergence of autoreactive T cell responses to apoptotic self-antigens [12], [13].

Successful priming of naïve CD4^+^ or CD8^+^ T cells results in the generation of both effector memory T (T_EM_) cells expressing various differentiation programs (type-1, -2, -17), according to the environment in which they are exposed [14]–[21], and central memory T (T_CM_) cells that promptly proliferate and generate new waves of effector cells on demand [22]–[24]. The transcription factor T-box-containing protein expressed in T cells (T-bet) is the master regulator of the type-1 cell differentiation program that is associated with the production of IFN-γ, which is required for the development of protective immune responses against intracellular pathogens [15]. GATA-binding protein 3 (GATA-3) controls the development of the type-2 cell lineage that is characterized by the production of IL-4, -5, and -13, which is critical for immunity against helminths and other extracellular pathogens [15]. Retinoid acid-related orphan receptor (ROR)-γt in mice and the human ortholog RORC in humans represent the master regulators of type-17 cell differentiation that leads to the production of IL-17, which is specifically required for protection against several types of extracellular and intracellular bacterial infections [14], [16]–[18]. All these (type-1, -2, -17) functions can elicit either protective or harmful effects, depending on whether they are executed by pathogen-specific or autoreactive T cells or whether the pathogen-specific are involved during an acute resolving infection or a chronic infection, respectively.

Here we used the hepatitis C virus (HCV) infection as a human model of acute infection that generally undergoes chronic progression to verify whether CD8^+^ T cells that are specific for apoptotic self-epitopes have a distinct effector type-1, -2, or -17 phenotype, to distinguish which of them is associated with the fate of a viral infection (recovery versus chronicity), and to ascertain the mechanisms whereby these responses are induced and maintained.

## Results

### Multispecificity of CD8^+^ T_EM_ cells to apoptotic epitopes correlates with infection chronicity

**Figure 1:**
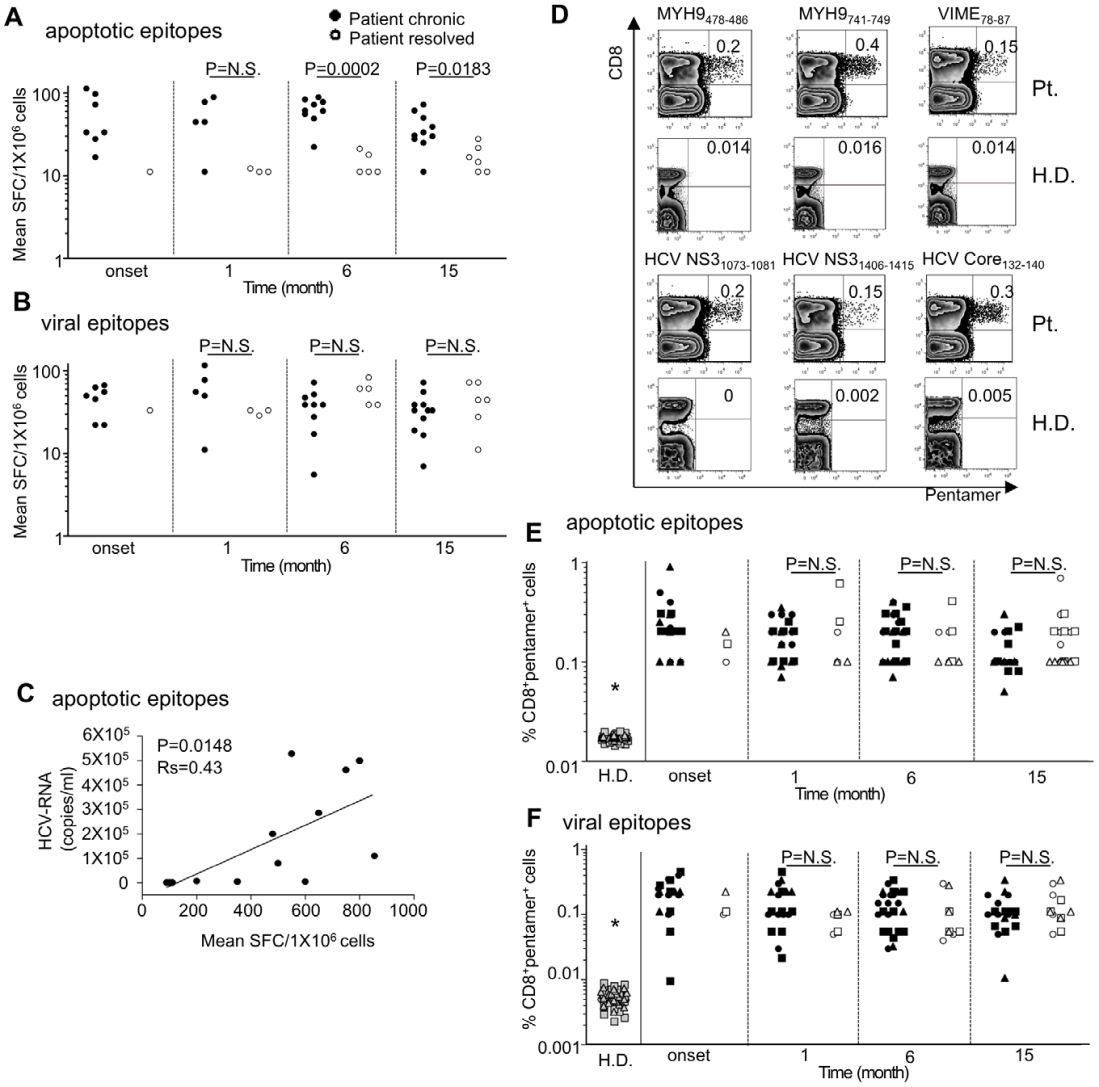
CD8^+^ T cell multispecificity to apoptotic epitopes in chronic HCV progression. (**A,B**) Mean number of spot-forming cells (SFCs) by fresh CD8^+^ T effector memory (T_EM_) cells (by ELISPOT assay) in response to pools (see **Tables S1A–C and S2A–E**) of apoptotic self-epitopes (A) or viral epitopes (B) in HLA-A2^+^ patients with acute HCV infection experiencing chronic infection (filled circles) or undergoing infection resolution (empty circles), evaluated at different time points of the infection. Each peptide pool contained 5 µg/ml each single peptide. Each circle represents a single patient. NS = not significant. (**C**) Representative correlation (6^th^ month after clinical onset) between the mean number of SFCs formed by fresh CD8^+^ T_EM_ cells in response to pools of apoptotic self-epitopes and the viral load (HCV-RNA copies/ml) in patients experiencing chronic infection or undergoing infection resolution. Statistical analysis was performed using non-parametric Spearman's test. (**D**) Representative flow cytometry analyses of PBMCs from a HLA-A2^+^ patient (Pt.) or a HLA-A2^+^ healthy donor (H.D.). Cells were then double-stained with a monoclonal antibody (mAb) to CD8 and pentamers expressing the indicated peptides of MYH9, VIME, HCV-NS3, or HCV-Core. Counterplot analyses show the percentage of CD8^+^pentamer^+^ cells. The percentage of cells is indicated in each quadrant. (**E,F**) Percentages of CD8^+^pentamer^+^ cells from20 HLA-A2^+^ H.D. and all HLA-A2^+^ patients studied (filled and empty symbols represent patients experiencing chronic infection or undergoing infection resolution, respectively) evaluated at different time points. In the panel E, circle symbols representMYH9_478–485_ pentamer specificity, square symbols represent MYH9_741–749_ pentamer specificity, and triangle symbols represent VIME_78–87_ pentamer specificity. In the panel F, circle symbols represent HCV-NS3_1073–1081_ pentamer specificity, square symbols represent HCV-NS3_1406–1415_ pentamer specificity, and triangle symbols represent HCV-Core_132–140_ pentamer specificity. NS = not significant. *The percentages of both apoptotic epitope- and viral epitope-specific CD8^+^pentamer^+^ cells from H.D. were significantly lower than those from patients (in any time point tested) (p<0.0001).

We analyzed longitudinally the responses of 18HLA-A2^+^ patients with acute HCV infection. The follow-up ranged from the onset of acute disease (**clinical onset**) to 15–24 months (the sixth month being considered the time of conversion from an acute to a chronic infection). Of the 18 patients, 6 patients had a self-limited infection and 12 patients exhibited a chronic evolution of infection (**Table 1**). Initially, the effector responses were determined by the capacity of freshly isolated CD8^+^ T cells from either HLA-A2^+^ patients or healthy controls to form IFN-γ spots (in an enzyme-linked immunospot [ELISPOT] assay) within 4 to 6 hours (h) of contact with nine pools of synthetic apoptotic peptides (**Table S1A–C**), eight pools of HCV genotype 1c, or genotype 2c peptides selected for their capacity to bind the HLA-A2 molecule [8], [25], or nine pools of overlapping peptides spanning the entire sequence of the HCV genotype 3a (**Table S2A–E**). The different HCV genotype-related peptides were matched with the viral genotype infecting the single patients. Each peptide pool was tested in triplicate. The synthetic apoptotic peptides used were prepared according to the sequence of caspase-cleaved proteins that had been previously identified by the proteomic analyses of apoptotic T cells (i.e., fragments of actin cytoplasmic 1 [ACTB], heterogeneous nuclear ribonucleo protein [ROK], lamin B1 [LAM1], non muscle myosin heavy chain 9 [MYH9], vimentin [VIME], or proteasome component C2 [PSA1]) [8]. We found that the apoptotic (but not the viral) epitope repertoire recognized by IFN-γ^+^CD8^+^ T_EM_ cells was significantly larger in patients undergoing chronic infection than in those undergoing recovery (**Fig. 1A,B** **and Fig. S1**). Interestingly, the mean number of IFN-γ spots promptly formed by CD8^+^ T_EM_ cells in response to each pool of apoptotic epitopes (but not viral epitopes) was directly correlated with the viral (plasma HCV-RNA) load, thus supporting the relationship between these responses and chronic evolution (**Fig. 1C**). None of the 21 HLA-A2^+^ healthy donors exhibited significant effector responses against any of the apoptotic or viral peptides *ex vivo* (data not shown). The HLA-restriction of these responses was demonstrated both by blocking responses with an appropriate anti-class I mAb and by determining that no response was observed in HLA-A2^−^ patients (data not shown).

**Table 1:** Clinical parameters of patients with acute HCV infection.

Pt	Genotype	Outcome	ALT (U/ml)	HCV-RNA (copies/ml)
1C	1b	Chronic	88	556000
2C	2a/2c	Chronic	695	3310
3C	3a	Chronic	1113	156000
4C	2a/2c	Chronic	600	915000
5C	1b	Chronic	227	600
6C	3a	Chronic	131	940000
7C	1b	Chronic	481	462000
8C	3a	Chronic	650	900000
9C	1a	Chronic	940	500000
10C	3a	Chronic	403	919000
11C	1b	Chronic	1200	640000
12C	1b	Chronic	1320	490000
1S	1b	Self-limited	2710	106000
2S	3a	Self-limited	1967	2120
3S	2a/2c	Self-limited	285	361000
4S	1a	Self-limited	1359	96100
5S	1b	Self-limited	2860	210200
6S	1b	Self-limited	2650	69500

Pt, patient; ALT, serum alanine aminotransferase (n.v., 0–40 U/ml).

### Mixed polyfunctional apoptotic epitope-specific CD8^+^ T cells in chronic HCV evolution

**Figure 2:**
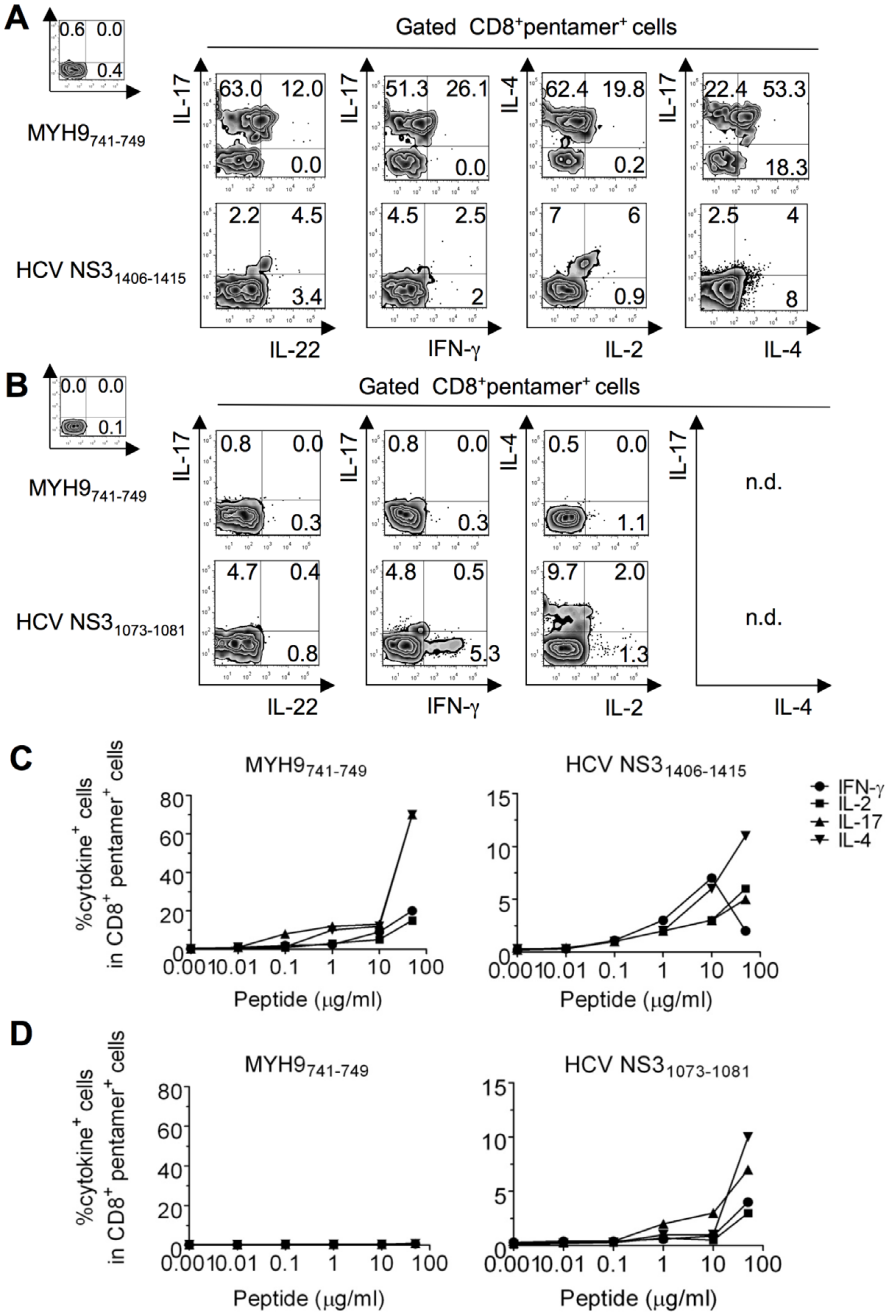
Polyfunctional CD8^+^ T_EM_ cells specific to apoptotic epitopes distinguish patients experiencing chronic infection. (**A,B**) Representative flow cytometry analyses of PBMCs from patients with acute HCV infection experiencing chronic infection (A) or undergoing infection resolution (B) that were stained with mAb to CD8 and pentamers complexed to the indicated apoptotic or viral epitopes. Cells were then stimulated with the relevant soluble peptides plus anti-CD28 mAb and processed for the detection of IL-17, IL-22, IFN-γ, IL-4, and IL-2 by ICS assay with the relevant mAbs. Counterplot analyses are gated on CD8^+^pentamer^+^ cells and show percentages of cytokine-producing cells. The percentage of cells is reported in each quadrant. The small histograms show ICS analyses of representative cytokine (IL-17, IL-22, IFN-γ, IL-4, or IL-2) production without antigenic stimulation by gated CD8^+^pentamer^+^ cells. (**C,D**) Peptide dose-response curves of cytokine-producing CD8^+^pentamer^+^ cells from patients with acute HCV infection experiencing chronic infection (C) or undergoing infection resolution (D). Cells were stained with the pentamers expressing the indicated peptides and stimulated for 6 h with the same soluble peptides. They were then processed for the detection of the different cytokines indicated by ICS assay. Values are shown with the background subtracted.

**Figure 3:**
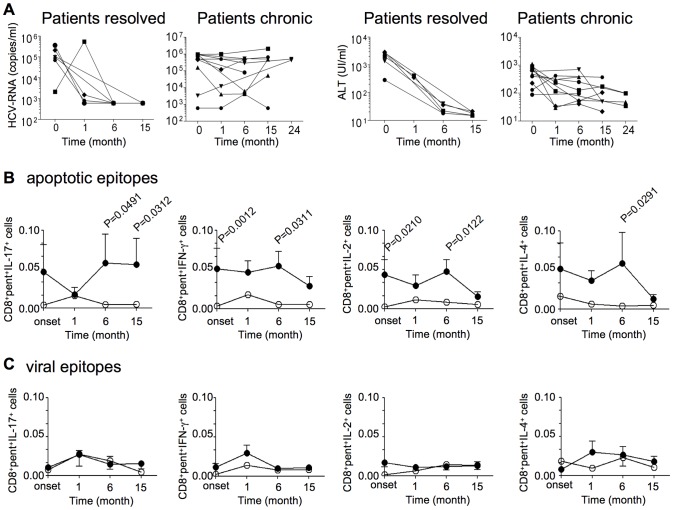
Polyfunctional CD8^+^ T_EM_ cells specific to apoptotic epitopes correlate with the chronic progression of HCV infection. (**A**) Kinetics of plasma HCV-RNA and ALT levels until 15–24 months from clinical onset of infection. Each symbol represents a single patient. Patients resolved: patients with acute HCV infection undergoing infection resolution; Patients chronic: patients with acute HCV infection undergoing chronic infection. (**B**) Kinetics of the means of all the CD8^+^pentamer^+^ (pent) cells specific to MYH9_478–485_, MYH9_741–749_, or VIME_78–87_ epitopes promptly producing the indicated cytokines in response to the relevant apoptotic epitopes. (**C**) Kinetics of the means of all the CD8^+^pentamer^+^ (pent) cells specific to HCV-NS3_1073–1081_, HCV-NS3_1406–1415_, or HCV-Core_132–140_ epitopes promptly producing the indicated cytokines in response to the relevant viral epitopes. The cells were calculated as percentage of cells expressing CD8, pentamer ligands, and cytokines in PBMCs. Values are shown with the background subtracted. Filled circles represent patients with acute HCV infection experiencing chronic infection; empty circles represent acute HCV-patients undergoing infection resolution.

**Figure 4:**
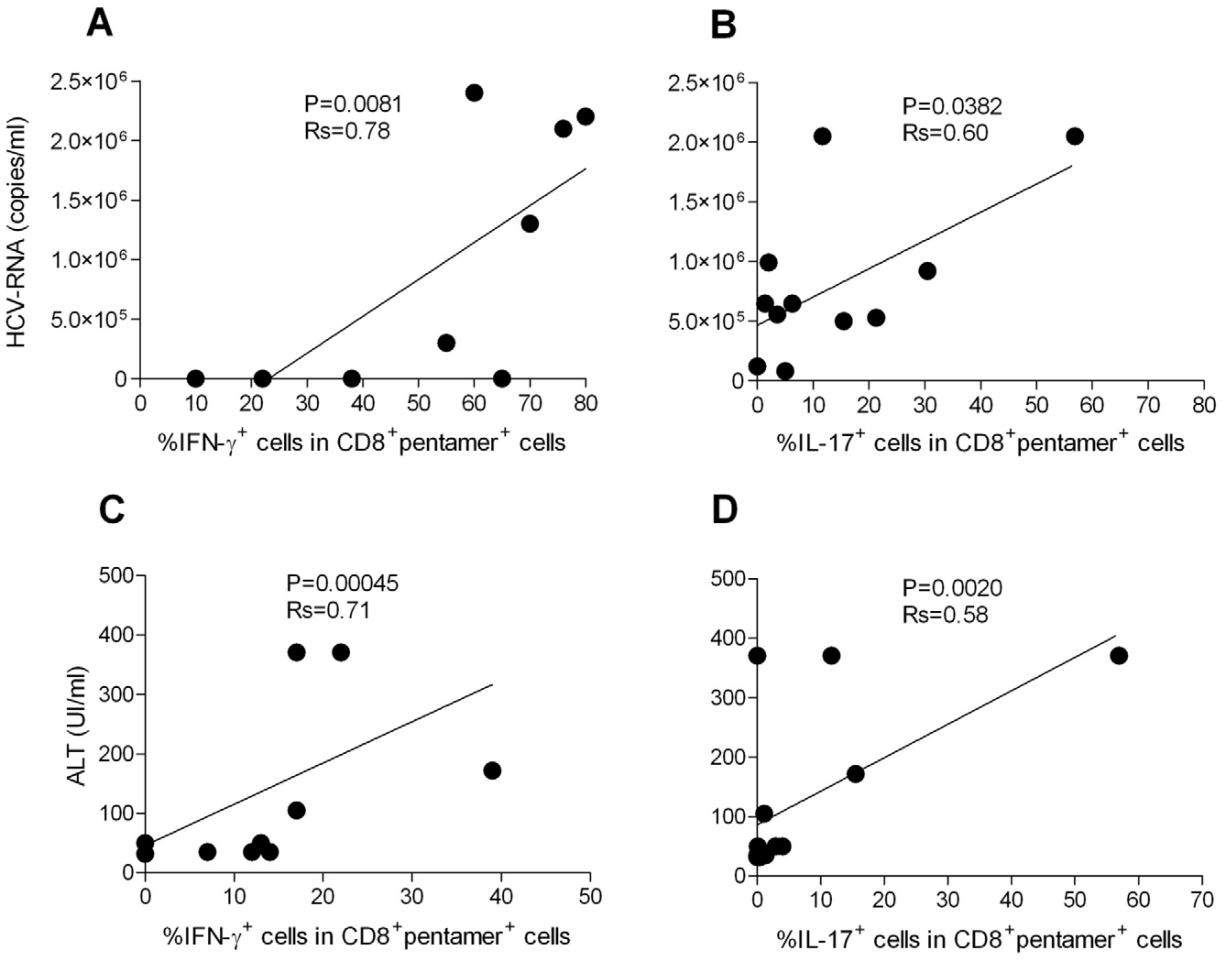
CD8^+^pentamer^+^ T_EM_ cells producing IFN-γ or IL-17 in response to apoptotic epitopes correlate with clinical parameters. (**A,B**) Correlation between the viral load (HCV-RNA copies) and CD8^+^pentamer^+^ T_EM_ cells producing IFN-γ (A) or IL-17 (B) in response to apoptotic epitopes in patients with acute HCV infection experiencing chronic infection or infection resolution. (**C,D**)Correlation between ALT and CD8^+^pentamer^+^ T_EM_ cells producing IFN-γ(C) or IL-17 (D) in response to apoptotic epitopes in patients with acute HCV infection experiencing chronic infection or infection resolution.

We enumerated specific CD8^+^ T cells directly in the peripheral blood of HLA-A2^+^patients or healthy donors by using pentamers of HLA-A*0201 molecules complexed to either apoptotic (MYH9_478–486_, MYH9_741–749_, VIME_78–87_) or viral epitopes (NS3_1073–1081_, NS3_1406–1415_, Core_132–140_) that had been previously identified as the most immunogenic among all patients tested (**Fig. 1D**). Control HLA-A*0201 pentamers complexed to a non-natural irrelevant peptide were unable to stain CD8^+^ T cells in all peripheral blood mononuclear cells (PBMCs) tested (data not shown). The pentamer values were significantly higher in both in patients experiencing chronic infection and in patients with self-limited infection (at all the time points tested) than in 20 HLA-A2^+^ healthy donors (**Fig. 1E,F**). However, in contrast to the ELISPOT assay showing frequencies of IFN-γ^+^CD8^+^Tcells specific to apoptotic peptides significantly higher in patients experiencing chronic infection than in patients with self-limited infection (**Fig. 1A**), the total frequencies of either apoptotic or viral epitope-specific CD8^+^ T cells, as detected by pentamers, did not differ between patients undergoing chronic or recovery evolution at all the time points tested (**Fig. 1D–F**). This difference may be explained by the finding that each single pentamer^+^ cell population can simultaneously contain (rare) naïve T cells, many T_CM_ cells and several types of T_EM_ cells with the same epitope specificity, as well as T cells with a “stunned phenotype” (representing the reducing capacity of cells to perform effector functions) [26], whereas ELISPOT assay only identifies IFN-γ+ cells in our system. To detect different effector functions within the CD8^+^pentamer^+^ T cells, we analyzed the frequencies of freshly isolated CD8^+^pentamer^+^ T cells that produced a wide array of cytokines (IL-17, IFN-γ IL-4, IL-2 within a few h of contact with the relevant peptides and optimal concentrations of anti-CD28 mAb, which served as a surrogate costimulatory signal. Irrelevant cytokine production was observed when either apoptotic epitope- or viral epitope-specific CD8^+^pentamer^+^ T cells of 20 HLA-A2^+^ healthy individuals were stimulated with this procedure (data not shown). Importantly, apoptotic epitope-specific CD8^+^pentamer^+^ T_EM_ cells promptly produced notable and sustained amounts of all the cytokines tested within a few h of contact with the relevant epitopes, much more in patients experiencing chronic infection than in those undergoing infection resolution (**Fig. 2A,B**), in all time points tested (Fig. **S2**). By contrast, the virus-specific CD8^+^pentamer^+^ T_EM_ cells produced lower amounts of the same cytokine in both categories of patients without any differences between them(**Fig. 2A,B** and **Fig.S3**). Peptide dose-response curves of cytokine-producing CD8^+^pentamer^+^ cells emphasized this difference (**Fig. 2C,D**). Time course analyses, performed longitudinally throughout the follow-up in all patients, revealed that the frequencies of polyfunctional apoptotic epitope-specific CD8^+^ T_EM_ cells were significantly higher in patients experiencing chronic infection (**Fig. 3A,B**). These responses were sustained over time in relation to the sustained viral load (HCV-RNA copies) and alanineaminotransferase (ALT) levels only in patients who evolved into chronic infection (**Fig. 3A,B**). Then, the majority of these cell frequencies, as well as the serum biomarkers of viral hepatitis, tended to decline considerably later in patients who evolved into chronic infection than in those resolving infection (**Fig. 3A,B**). By contrast, no substantial difference was revealed in the time course of the virus-specific effector response between the two categories of patients (**Fig. 3C** **, Fig. S3**). Notably, the polyfunctional responses in the majority of patients were maintained by the parallel presence of different antigen-specific CD8^+^ T cell subsets, each of which produced a single cytokine in all time-points tested (mixed polyfunctional populations) (**Fig.S4A,B**). Therefore, the minority of patients showed cells simultaneously producing significant amounts of IFN-γ and IL-17 (type 1/17 cells), or cells simultaneously producing significant amounts of IL-17 and IL-4 (type 2/17 cells) (**Fig. 2A,B** and **Fig. S4A,B**). Importantly, the frequencies of CD8^+^pentamer^+^ T_EM_ cells promptly producing IFN-γ or IL-17 in response to the relevant apoptotic epitopes, but not to the viral epitopes (data not shown), were directly correlated with the plasma viral load or the serum ALT levels(**Fig. 4A–D**).

### Cross-presentation of apoptotic T cells *ex vivo*

**Figure 5:**
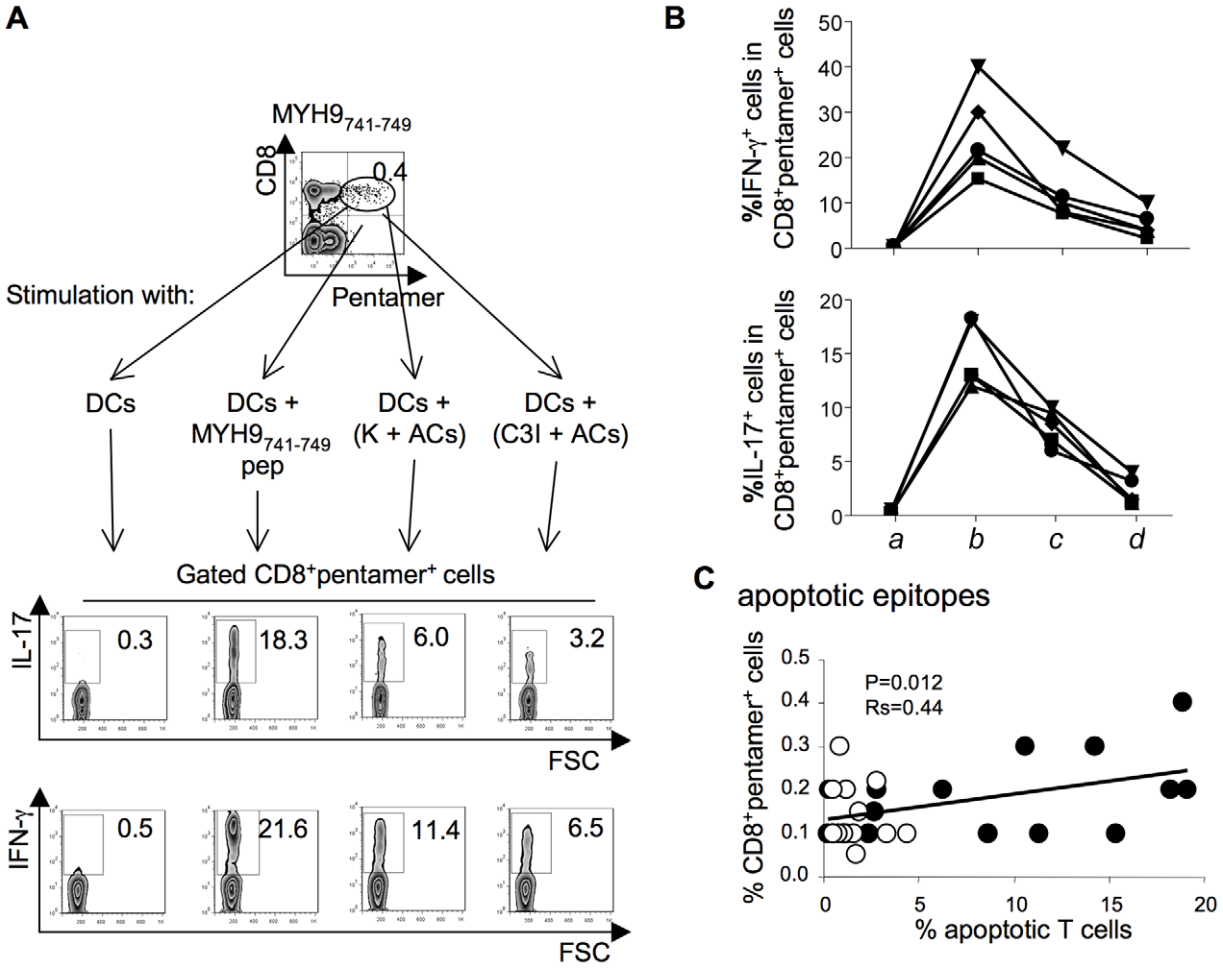
CD8^+^ T_EM_ cells specific to apoptotic epitopes are activated by apoptotic epitopes naturally processed and cross-presented by DCs. (**A**) One representative of five experiments in which PBMCs from one patient with acute HCV infection were double-stained with mAb to CD8 and pentamers complexed with the indicated apoptotic epitope, and cultured with autologous DCs that had been pulsed or not with the relevant soluble peptide, apoptotic cloned T cells (ACs = apoptotic cells), ACs previously treated with a negative caspase control (K = Z-FA-FMK), or ACs previously treated with the caspase 3 inhibitor (C3I = z-devd-fmk). After 6 h, CD8^+^pentamer^+^ cells were tested for their capacity to produce the different cytokines indicated by the ICS assay. Counterplots are gated on CD8^+^pentamer^+^ cells and show percentages of the different cytokine-producing cells in each quadrant. (**B**) Cumulative experiments in 5 independent patients, performed as described in (A), showing the percentages of cells producing the indicated cytokines in CD8^+^pentamer^+^ cells in response to the following stimuli: autologous DCs alone (*a*); DCs that had been pulsed with the MYH9_741–749_ peptide (*b*); DCs that had been pulsed with ACs, previously treated with a negative caspase control (Z-FA-FMK) (*c*); or DCs that had been pulsed with ACs previously treated with the caspase 3 inhibitor (Z-DEVD-FMK) (*d*). Each symbol represents a single patient. (**C**) Correlation (analysed at the 15^th^ month after clinical onset) between the percentage of CD8^+^pentamer^+^ cells specific to apoptotic epitopes and the percentage of circulating apoptotic T cells in patients with acute HCV infection experiencing chronic infection (filled circles) or infection resolution (empty circles). Statistical analysis was performed using non-parametric Spearman's test.

Fresh apoptotic epitope-specific CD8^+^pentamer^+^ T_EM_ cells promptly produced IFN-γ or IL-17 *ex vivo* within a few h of contact with DCs that had been pulsed with apoptotic T cells (i.e., through the cross-presentation mechanism) (**Fig. 5A,B**). The cross-presentation resulted in a marked decrease in IFN-γ or IL-17 production when apoptotic cells had been previously treated with a selective caspase-3 inhibitor (C3I) (**Fig. 5A,B**). This phenomenon was confirmed in five independent patients (**Fig. 5B**). DCs alone, despite known to endogenously express high levels of the ubiquitous (long-lived) cellular proteins (vimentin, non-muscle myosin, actin, heterogeneous nuclear ribonucleoprotein, lamin B1…) (14),were unable to directly stimulate the related specific CD8^+^ T cells (**Fig. 5A,B**).The frequencies of apoptotic epitope-specific CD8^+^ T cells (but not those of viral epitope-specific CD8^+^ T cells [data not shown]) correlated with the number of circulating apoptotic T cells (**Fig. 5C**).The percentage of apoptotic T cells in PBMCs was significantly higher in patients than in the 20 healthy donors tested (11.0±7.7 versus 3.9±3.9; *P*<.001).

### Flexibility of type-17 CD8^+^ T_EM_ cell responses to apoptotic epitopes

**Figure 6:**
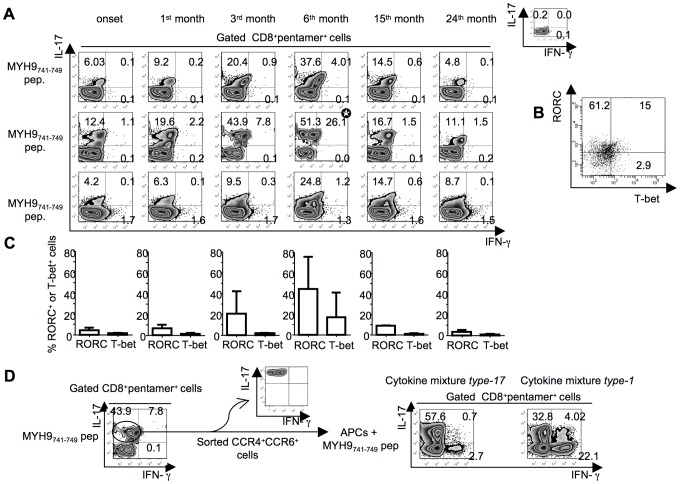
Functional flexibility of fresh IL-17–producing CD8^+^ T cells specific to apoptotic epitopes. (**A**) PBMCs from three independent patients with acute HCV infection were stained with mAb to CD8 and pentamers complexed with the indicated apoptotic peptide. Cells were stimulated with the relevant soluble peptide plus anti-CD28 mAb for 6 h and then processed for the detection of IL-17 and IFN-γ by ICS assay with the relevant mAbs. Counterplot analyses are gated on CD8^+^pentamer^+^ cells and show percentages of cytokine-producing cells. The percentage of cells is reported in each quadrant. The small histograms show ICS analyses of representative cytokine (IL-17, or IFN-γ) production without antigenic stimulation by gated CD8^+^pentamer^+^ cells. (**B**) Representative flow cytometry analysis in which cells were stimulated as previously described and processed for the detection of IL-17, IFN-γ, RORC, and T-bet by ICS assay with the relevant mAbs. Analysis gated on CD8^+^pentamer^+^IL-17^+^ IFN-γ^+^ cells (signed by the star symbol in the panel A) show percentages of RORC- and Tbet-expressing cells. The percentage of cells is reported in each quadrant. (**C**) Mean percentages of RORC^+^ or T-bet^+^ cells detected in all the timely followed CD8^+^pentamer^+^ cells producing IL-17 or IFN-γ upon antigen stimulation. (**D**)IL-17–producing CD8^+^ T cells specific to apoptotic epitopes efficiently convert to IFN-γ producing cells *in vitro*. One representative of three experiments in which PBMCs from patients with acute HCV infection were stained with mAb to CD8 and pentamers complexed with the indicated apoptotic peptide, stimulated with the relevant soluble peptide plus anti-CD28 mAb for 6 h, and processed for the detection of IL-17 and IFN-γ by ICS assay with the relevant mAbs. Counterplot analyses are gated on CD8^+^pentamer^+^ cells and show percentages of cytokine-producing cells. The percentage of cells is reported in each quadrant. Cells producing IL-17 were sorted by using anti-CCR6 and anti-CCR4 mAbs and FACSAria processing (the purity of IL-17^+^ cell population was evaluated by stimulating CCR6^+^CCR4^+^ cells with anti-CD3 and anti-CD28 mAbs). CCR6^+^CCR4^+^ cells were re-stimulated with the same apoptotic epitope and autologous APCs in the presence or absence of either the cytokine mixture *type-17* (IL-1β, IL-6, IL-23 and TGF-β) polarizing toward the type-17 cell phenotype or the cytokine mixture *type*-1 (IFN-γ and IL-12) polarizing toward the type-1 cell phenotype. After 10–12 d of culture in IL-2 conditioned medium, cells were stained with pentamers expressing MYH9_741–749_ peptide, further antigen-stimulated as previously described, and tested for their capacity to produce IL-17 and IFN-γ by ICS assay.

To verify if type-17 CD8^+^ T_EM_ cells specific to apoptotic self-antigens in the long run acquire functional plasticity *in vivo* [15], [18], [27]–[29], we monitored (from the clinical onset of infection up to 24 months) selected patients showing a notable number of CD8^+^ T_EM_ cells promptly producing IL-17 within few h of contact with the relevant apoptotic epitopes at the clinical onset. During the course of the follow-up, the frequency of type-17 CD8^+^ T_EM_ cells exhibited a progressive increase, followed by the emergence of type-1/17 cells in response to apoptotic epitopes (**Fig. 6A**). These responses were associated with both the maintenance of the type-17 transcription factor RORC and the appearance of the type-1 transcription factor T-bet (**Fig. 6B,C**). This scenario was observed both in the 3 patients showing type-17 CD8^+^ T_EM_ cells specific for the MYH9_741–749_ epitope (**Fig. 6**), and in additional 3 patients showing type-17 CD8^+^ T_EM_ cells specific for different self-epitopes epitope (data not shown). By contrast, representative fully polarized type-1 CD8^+^ T_EM_ cells strictly maintained this phenotype throughout the follow-up period in all patients studied (**Fig. S5**online). To determine whether antigen-specific type-17 CD8^+^ T cells can reprogram their phenotype and convert into type-1/17 CD8^+^ T_EM_ cells *in vitro*(situation which may mimic the type-17 conversion into type-1/17 phenotype *in vivo*), we used anti-CCR6 and anti-CCR4 mAbs [30] to sort IL-17–producing cells from antigen-stimulated CD8^+^ T cells (purity >98% type-17 pentamer^+^CD8^+^ T cells) (**Fig. 6D**). These cells were then restimulated *in vitro* with irradiated autologous PBMCs (acting as antigen-presenting cells [APCs]) that had previously been pulsed with the relevant peptide in the presence of either a mixture of IFN-γ and IL-12 (polarizing toward the type-1 phenotype) or a mixture of TGF-β, IL-6, IL-23, and IL-1β (polarizing toward the type-17 phenotype) [14], [31]. After 10–12 days of culture in IL-2 conditioned medium, the cells were tested for their capacity to produce both IL-17 and IFN-γ in response to the peptide plus APCs. Interestingly, CD8^+^ T cells that had been cultured in the presence of the type-17 polarizing cytokines maintained or increased the original type-17 phenotype, whereas CD8^+^ T cells that had been cultured in the presence of the type-1 polarizing cytokines switched (in a notable proportion) toward the type-1 phenotype (**Fig. 6D**).

### Polyfunctional autoreactive CD8^+^ T cells are modulated by PD-1

To understand why the self-epitope-specific cells of patients undergoing resolution display significantly lower polyfunctional functions than patients experiencing chronic infection (**Fig. 2A–D** and **Fig. 3A,B**), we performed a series of functional experiments. First, we ruled out the possibility that apoptotic epitope-specific CD8^+^ T cells from patients undergoing recovery expressed intrinsic defects of effector cell functions. Indeed, apoptotic epitope-specific CD8^+^ T cells from the two categories of patients yielded similar cytokine responses after stimulation by polyclonal mitogens (i.e., phorbol 12-myristate 13-acetate [PMA] and ionomycin [iono]) (**Fig. S6**). Second, the majority of CD8^+^pentamer^+^ T cells (both apoptotic epitope-specific and viral epitope-specific) in both categories of patients were either CD45RO^+^CD127^+^ (T_CM_ cells) or CD45RO^+^CD127^−^ (T_EM_ cells) (**Fig. S7A,B**). This finding suggested that the CD127^−^CD8^+^ T_EM_ cells, which promptly produced the vast array of cytokines tested within a few hours of contact with the relevant peptides (**Fig. S7C**), were likely derived from the CD8^+^ T_CM_ cells rather than naïve cells in both categories of patients *in vivo*.

**Figure 7:**
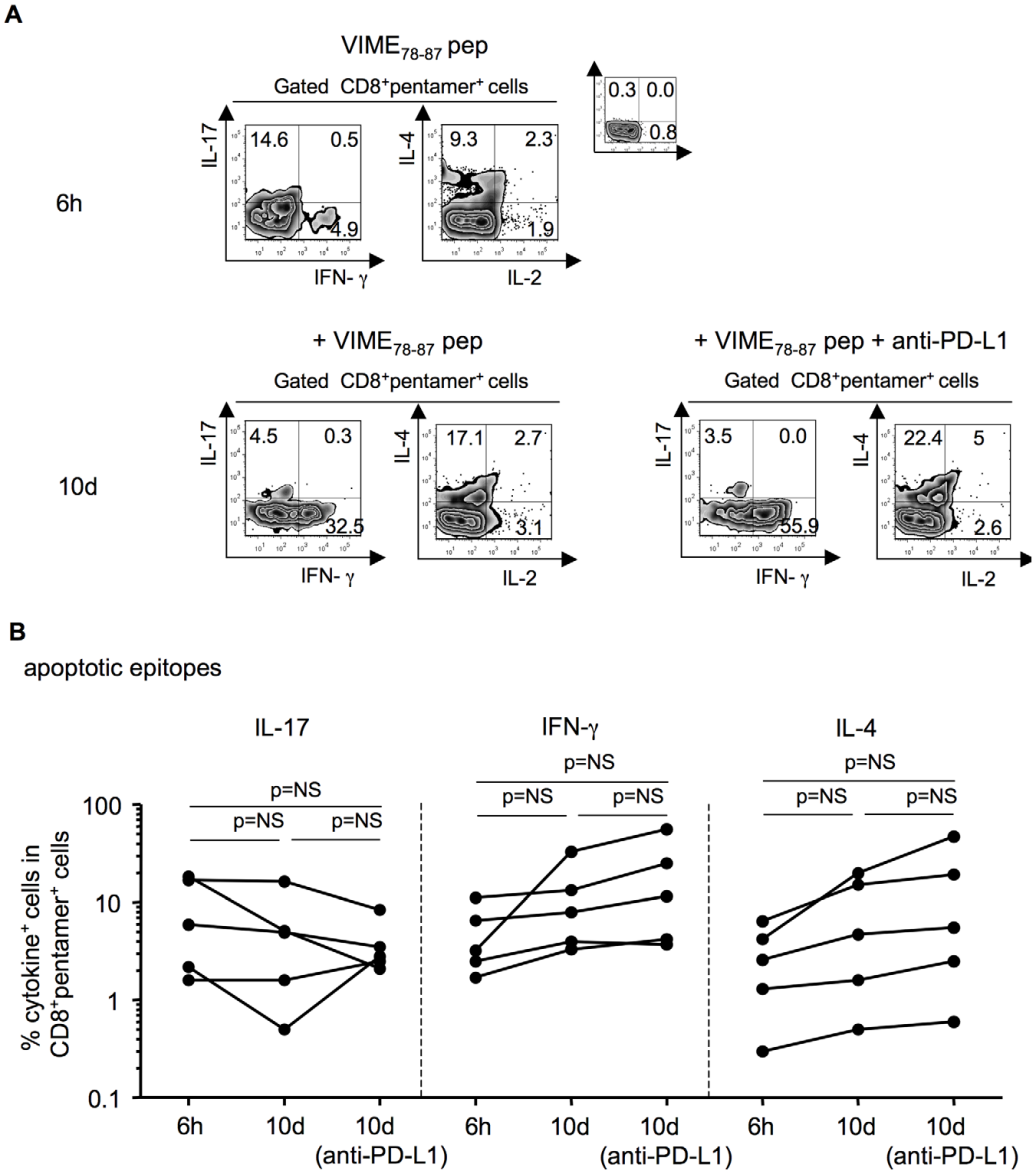
Tuning of apoptotic epitope-specific CD8^+^ T cells by PD-1. (**A**) Representative experiment in which PBMCs from a patient with acute HCV infection were stained with mAb to CD8 and the pentamer complexed with the indicated apoptotic epitope. Cells were then stimulated with the soluble form of the indicated peptide and anti-CD28 mAb in the presence or absence of a blocking antibody to PD-L1 or an isotype control. After 6 h and 10 d of culture, cells were processed for the detection of the indicated cytokines by ICS assay with the relevant mAbs. (**B**) Cumulative experiments, performed as described in (a), showing the percentages of cells producing the indicated cytokines in CD8^+^pentamer^+^ cells, evaluated at different time points from the stimulation with the apoptotic peptides in the presence or absence of mAb to PD-L1. NS = not significant.

Then, we evaluated whether the apoptotic epitope-specific CD8^+^ T_EM_ cells were less polyfunctional in patients undergoing infection resolution because they were conditioned by a more severe programmed death (PD)-1-dependent exhaustion [32] in comparison to those from patients experiencing chronic infection. We found a similar PD-1 expression in apoptotic epitope-specific CD8^+^ T cells between patients undergoing infection resolution and those experiencing chronic infection (**Fig. S8A**).This result might argue against the possibility of a more severe PD-1-dependent exhaustion of apoptotic epitope-specific CD8^+^ T_EM_ cells from patients resolving infection. However, we cannot exclude that the two groups of patients might express different levels of PD-1 ligands (i.e., in inflamed liver) that might provide different threshold of PD-1 dependent exhaustion *in vivo*. PD-1 upregulation was also shown in HCV-specific CD8^+^ T cells without any significant difference between the two categories of patients (**Fig. S8B**). To determine the functional capacity of PD-1 expression, we first selected PBMCs containing either viral epitope-specific PD-1^+^CD8^+^ T cells or apoptotic epitope-specific PD-1^+^CD8^+^ T cells from patients undergoing infection resolution or those experiencing chronic infection. In the presence or absence of a blocking anti-PD-L1 mAb or isotype control mAb *in vitro*, these cells were stimulated with the relevant peptide and anti-CD28. After 10 days of culture in IL-2-conditioned medium, cells were double-stained with the appropriate pentamers and anti-CD8 mAb, stimulated or not with autologous APCs plus the peptide, processed for intracellular cytokine staining (ICS) with mAbs to IFN-γ, IL-17, and IL-4, and analyzed by flow cytometry. Apoptotic epitope-specific pentamer^+^CD8^+^ T cells produced notable amounts ofIFN-γ or IL-4 after 10 d of stimulation, and even more in the presence of a blocking anti-PD-L1 mAb (**Fig. 7A**). By contrast, IL-17 production under the same conditions failed to increase but rather decreased during the 10 d of antigen stimulation *in vitro*, irrespective of the presence of anti-PD-L1, emphasizing the possible functional instability of this cell population (**Fig. 7A**). Cumulative experiments with PBMCs from a total of eight patients confirmed that the PD-1/PD-L1 blockade resulted in an increase of IFN-γ or IL-4 production by both apoptotic epitope-specific pentamer^+^CD8^+^ T cells (**Fig. 7B**) and viral epitope-specific pentamer^+^CD8^+^ T cells (data not shown), whereas the production of IL-17 was not affected. Importantly, the degree of increase in the responses exhibited by both apoptotic epitope-specific pentamer^+^CD8^+^ T cells and viral epitope-specific pentamer^+^CD8^+^ T cells was virtually the same between patients undergoing infection resolution and those evolving into chronic infection (data not shown). Taken together, these findings suggest the following. First, the apoptotic epitope-specific PD-1^+^CD8^+^ T cell responses are gently modulated by PD-1 because they are highly polyfunctional in patients experiencing chronic infection *ex vivo*. Second, the PD-1 blockade does not seem a principal cause of the decreased responsiveness exhibited by apoptotic epitope-specific CD8^+^ T cells from patients undergoing infection resolution in comparison to responses from patients who have developed a chronic infection, given that the degree of increase in the effector responses upon PD-1/PD-L1 blockade was very similar between the two categories of patients. However, we cannot exclude that other PD-1 ligands or the differential PD-L1 expression by inflamed liver can intervene in favoring T cell exhaustion or dysfunction *in vivo*, thus explaining the decreased responsiveness exhibited by apoptotic epitope-specific CD8^+^ T cells from patients undergoing infection resolution [33].

### Linking TCR avidity of apoptotic epitope-specific CD8^+^ T cells to infection outcome

**Figure 8:**
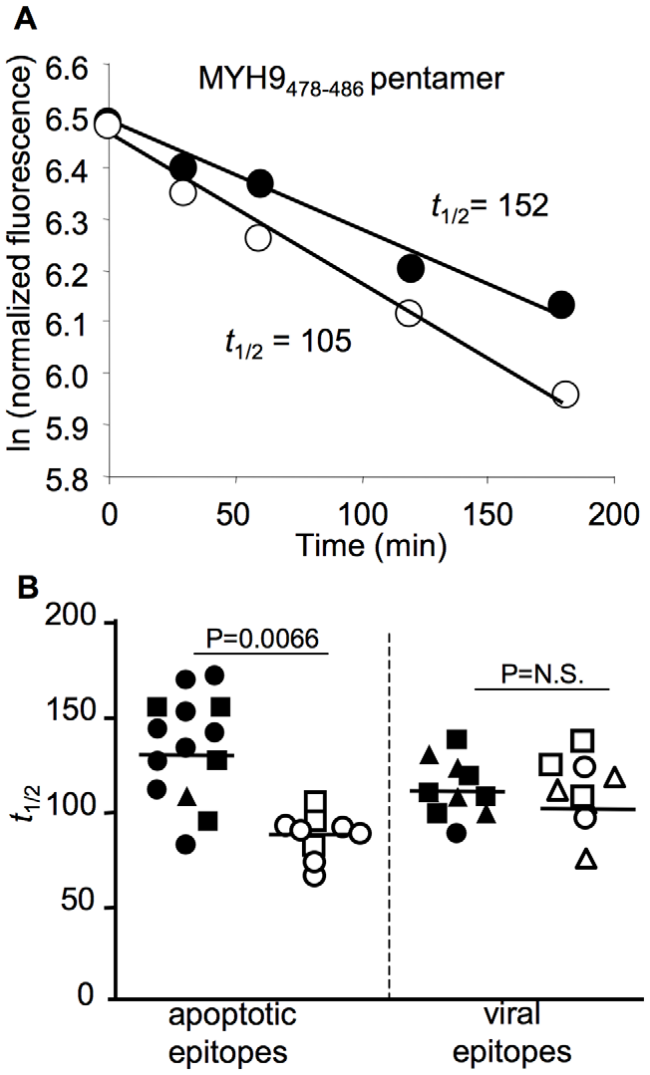
Decay kinetics of pentamer staining for CD8^+^ T cells. (**A**) Representative decay plot of the natural logarithm of the normalized fluorescence versus time after anti-HLA-A2 mAb addition for fresh PBMCs stained with pentamers that were complexed with the indicated apoptotic epitope. The *t*_1/2_ represents the staining half-times for the pentamers to CD8^+^ T cells. Filled or empty circles represent PBMCs from patients with acute HCV infection experiencing chronic infection or undergoing infection resolution, respectively. (**B**) The *t*_1/2_ for the pentamers (complexed with apoptotic or viral epitopes) binding CD8^+^ T cells from patients with acute HCV infection experiencing chronic infection (filled symbols) or undergoing infection resolution (empty symbols). Apoptotic epitopes: circle symbols represent the MYH9_478–485_ pentamer specificity, square symbols represent MYH9_741–749_ pentamer specificity, and triangle symbols represent VIME_78–87_ pentamer specificity. Viral epitopes: circle symbols represent HCV-NS3_1073–1081_ pentamer specificity, square symbols represent HCV-NS3_1406–1415_ pentamer specificity, and triangle symbols represent HCV-Core_132–140_ pentamer specificity.

Finally, we hypothesized that differences in T cell receptor (TCR) avidity might account for the different apoptotic epitope-specific CD8^+^ T cell responsiveness between patients experiencing chronic infection and those undergoing infection resolution. To assess TCR avidity, we evaluated the dissociation kinetics of peptide/HLA-A*0201 pentamer binding to antigen-specific CD8^+^ T cells that were isolated from the two groups of patients [34]. Specifically, we stained fresh CD8^+^ T cells with saturating amounts of HLA-A*0201 pentamers that were complexed to either apoptotic or viral epitopes and an anti-CD8 mAb for 45 min at room temperature. Cells were then washed and incubated at 4°C with saturating amounts of an anti-HLA-A2 mAb to prevent rebinding of pentamers during the pentamer dissociation assay. The rate of decay was measured by flow cytometry at appropriate time points. We obtained linear decay plots of the natural logarithm of the normalized fluorescence versus time in all experiments performed, indicating that the pentamer decay was occurring stochastically and that the resulting pentamer staining half-lives(*t*_1/2_) should be proportional to the *t*_1/2_ of respective pentamer/TCR complexes (**Fig. 8A**). The *t*_1/2_ for apoptotic epitope-complexed pentamer staining to fresh CD8^+^ T cells from patients experiencing chronic infection was significantly longer than the decay of pentamer staining to CD8^+^ T cells from patients undergoing infection resolution (**Fig. 8A,B**). By contrast, the *t*_1/2_ for viral epitope-complexed pentamers to CD8^+^ T cells did not differ between patients experiencing chronic infection and those undergoing infection resolution (**Fig. 8A,B**). Control experiments in which HLA-A*0201 pentamers were complexed to a non-natural irrelevant peptide showed that the *t*_1/2_ for staining to CD8^+^ T cells was undetectable (data not shown).

## Discussion

Here we demonstrate for the first time that the multispecificity, magnitude, and polyfunctional (type-1, -2, -17)strength of CD8^+^ T_EM_ cell responses directed to apoptotic self-epitopes were wide and robust during the acute phase of HCV infection, particularly in patients experiencing chronic progression compared with those undergoing infection resolution. The responses were directly correlated with the plasma viral load, the serum ALT levels or the number of circulating apoptotic T cells, and were then sustained over time in relation to the viral persistence. Our parallel study still in progress indicates that similar autoreactive CD8^+^ T cell responses in chronically infected patients are recruited in the inflamed livers (**Fig. S9**), are related with the signs of hepatic damage, and decrease in relation with the decline or the disappearance of the viral load upon antiviral therapy (interferon plus ribavirin^@^) (data not shown). Altogether these results suggest that strong CD8^+^ T cell responses against apoptotic self-epitopes arise and are maintained in HCV infection and may potentially contribute to the liver immunopathology through the production of high levels of inflammatory cytokines.

Recently, several models of chronic viral infection demonstrated that virus-specific CD4^+^ or CD8^+^ T cells producing elevated levels of IL-17 correlated with either viral persistence or a wasting syndrome with a multiple organ neutrophil infiltration [20], [35], [36]. Currently, our data suggest that the emergence of high frequencies of mixed autoreactive CD8^+^ T cells producing a broad array of cytokines (including IL-17) is prominent in patients undergoing chronic progression in the human model of acute HCV infection. By contrast, the frequencies of virus-specific effector cells (producing the different cytokines analyzed) were extremely low as compared with the apoptotic epitope-specific. Our data are coherent with the majority of studies revealing that the magnitude of HCV-specific CD8^+^ T cell effector responses does not correlate with the clinical or viral outcome in acute HCV infection [37]. In particular, HCV-specific CD8 T cells have been reported to express increased levels of PD-1 and an exhausted phenotype (weak proliferation, IFN-γ production, and cytotoxicity) [37]–[41].Although depletion studies in the chimpanzee model are consistent with a role of CD8^+^T cells as primary effector of protective immunity [42], studies in natural HCV infection were unable to find clear correlations between HCV-specific CD8^+^ T cell responsiveness and outcome of infection [37]–[41], [43], [44]. It is possible that the mechanisms that control HCV in the long term lie not exclusively on these conventional functions, but they are also displayed by some other subset of immune mediators, including HCV-specific antibodies [45]or CD4^+^ T cells [26], [40], [46].Consistent with this finding, *in vivo* depletion of CD4^+^ T cells from HCV-recovered chimpanzees abrogates protective CD8^+^ T cell–mediated immunity upon rechallenge [47], which suggests that CD4^+^ T cell help is required for the generation and maintenance of protective CD8^+^ T cells. Therefore, the viral immunological correlates of infection should be detected by multiparametric analyses (antibody, CD4^+^, CD8^+^ responses…) rather than individual analysis that may underestimate the multiple immunological variables related to infection outcome. In this respect, our study suggests that the apoptotic epitope- more than the virus-specific CD8^+^ cell responses discriminate patients with different infection outcome.

The observation that cross-presentation of apoptotic T cells by DCs requires caspase-dependent cleavage of apoptotic self-antigens to promptly activates specific CD8^+^ T_EM_ cells *ex vivo* indicates that this mechanism might be operative in the induction of the related polyfunctional autoreactive responses *in vivo*. This possibility is emphasized by the finding that the frequencies of apoptotic epitope-specific CD8^+^ T cells correlated with the number of circulating apoptotic T cells. Consistently with our previous observations (14), cross-presentation of apoptotic cells plays a key role in activating autoreactive CD8^+^ T cells, as caspase-dependent cleavage of cell-associated (long-lived) proteins (such as vimentin, non-muscle myosin, actin, heterogeneous nuclear ribonucleoprotein, lamin B1…) is required to efficiently target the related fragments to the processing machinery of DCs. By contrast, live DCs alone, despite known to express the whole form of the same ubiquitous (long-lived) cellular proteins(14), are unable to stimulate the related specific CD8^+^ T cells by direct presentation mechanism, likely because they do not possess the caspase-cleavage program required for the presentation of these proteins (14).Collectively, these data suggest that these autoreactive CD8^+^ T cells may perform their functions through the by-stander effect of the pro-inflammatory cytokines upon cross-presentation of apoptotic cells rather than by the direct killing of cells endogenously expressing the related self-antigens. The strong production of IFN-γ and IL-17 may favor the triggering of recruitment of inflammatory cells, which contribute to the immunopathology [48]–[50].Our study provides a possible explanation for why the enormous expansion of activated T cells, during persisting viral infections, is only minimally attributable to virus-specific T cells [8]. Inflamed tissues (including the HCV-infected liver) are generally infiltrated by several billions of activated lymphocytes and the rate of apoptotic cells derived from them by far exceeds that originated by the turn-over of epithelial cells (i.e., hepatocytes) [51]. The demonstration that apoptotic cells derived from activated T cells (in contrast to those derived from epithelial cells) are CD40L^+^ and then condition CD40^+^ DCs to prime T cells [12], [13], suggest that they are the most important source of apoptotic self-antigens capable to cross-prime CD8^+^ T cell responses in an inflamed microenvironment. However, we cannot exclude that also apoptotic hepatocytes may amplify this phenomenon in an inflammatory context, because they might potentially generate the same caspase-cleaved antigenic fragments described in apoptotic T cells [8], and be cross-presented by DCs.

Recent data have clearly stressed the importance of infections in inducing and maintaining autoimmunity [52]. In particular, the initial emergence of apoptotic antigen-specific T cells in acute HCV infection may be dependent on virus-specific T cells that can provide the first waves of apoptotic substrates, upon performing their effector function. This mechanism may be maintained in patients evolving towards the viral persistence, and be further amplified by the apoptotic antigen-specific T cells themselves providing further waves of apoptotic antigens. Additional studies, even in appropriate experimental models, are required to ascertain the role of these autoreactive responses in the chronic evolution of infection. Moreover, it could be of interest to investigate if an expansion of mucosal associated invariant type-17 CD8^+^ T cells may participate in the high IL-17 production [53], as well as if other additional cytokines including IL-10 [54] may increase the polyfunctionality of apoptotic epitope-specific CD8^+^ T cells.

IL-17 production can account for the transient intra-hepatic infiltration of neutrophils found only in the early phase of acute HCV infection [55]. Then, these responses timely decline likely through the simultaneous presence of autoreactive type-1, -2, and -17 CD8^+^ T_EM_ cells, which may regulate each other, and even the capacity of type-17 CD8^+^ T_EM_ cells to express a certain degree of plasticity [15], [18], [27]–[29], and to convert to type 1/17 CD8^+^ T_EM_ cells. Therefore, the environmental setting during an acute inflammatory disease seems to be addressed to guarantee the coexisting polarization of type-1, -2, -17, -1/17 CD8^+^ T_EM_ cells, and even type-2/17 CD8^+^ T_EM_ cells, likely to limit excessive damage by fine-polarized type-1 or type-17 CD8^+^ T_EM_ cells. In this context, it is intriguing the observation that the polyfuctional autoreactive CD8^+^ T cells express high PD-1 levels *in vivo* and are limited only partially by the inhibitory PD-1 capacity *in vitro*. A possible explanation of this is that these autoreactive CD8^+^ T cells are primed later and thus submitted to a less prolonged antigenic stimulation than the virus-specific, that in contrast show a profound exhaustion in patients with HCV infection [32]. Recently, the persistent antigenic stimulation has been demonstrated to cause down regulation of T-bet, which results in more severe exhaustion of virus-specific CD8^+^ T cells [56]. However, the tuning of autoreactive CD8^+^ T_EM_ cell functions by PD-1, as well as the high frequencies of apoptotic epitope-specific CD8^+^ T_EM_ cells producing IL-4, may contribute to limit excessive functional responses over time [19], [54], [57], [58].In support of this hypothesis, our study in patients with long-term chronic HCV infection demonstrates that liver-infiltrating CD8^+^ T_EM_ cells specific to apoptotic self-epitopes produce levels of cytokines significantly lower than patients with acute hepatitis (data not shown).

An important facet of our findings is that they demonstrate a link between the TCR avidity of autoreactive CD8^+^ T cells and the difference in the responsiveness of apoptotic epitope-specific CD8^+^ T cells exhibited by patients experiencing chronic infection and those undergoing infection resolution. The dissociation kinetics of peptide/HLA-A*0201 pentamer binding to antigen-specific CD8^+^ T cells clearly demonstrated that the *t*_1/2_ for apoptotic epitope-complexed pentamer staining to CD8^+^ T cells from patients experiencing chronic infection was significantly longer than the decay of pentamer staining from patients undergoing infection resolution. The *t*_1/2_ for pentamer staining was detected on freshly isolated CD8^+^ T cells, suggesting that TCR avidity measured by this system likely reflects what occurs *in vivo*. In the original study, this methodological approach made it possible to postulate that T cells with TCRs that bind peptide/MHC complexes for a longer duration are selectively preserved in comparison to T cells that express TCRs with lower avidity [34]. Recent studies suggested that in response to different microbial infections, initially naïve T cells with a wide range of avidity are efficiently recruited and expanded [59], [60]; subsequently, those with lower avidity undergo premature contraction, whereas those with higher avidity are selected because of a more prolonged expansion and correlate with protection [59], [60]. Our results provide an additional challenge to this model, and demonstrate that the TCR avidity of autoreactive CD8^+^ T cells specific for apoptotic self-epitopes was significantly higher in patients undergoing chronic infection than in those resolving infection. The selection of the autoreactive CD8^+^ T cells with higher avidity likely occurs because of a sustained stimulation by apoptotic antigens [8]. By contrast, lower avidity CD8^+^ T cells in the presence of weaker stimuli would undergo rapid contraction, as seen in the peripheral blood of patients with self-limited HCV infection. The viral persistence may provide the conditions that influence the availability of sustained apoptotic antigenic stimuli. However, our model does not exclude the possibility that cross-reactive CD8^+^ T cells, even expressing dual TCR [61], may intervene in this process.

Finally, our results may provide an important platform for the design of innovative therapeutic strategies to re-engineer protective immune responses in persisting infections. In addition, further studies will ascertain whether polyfunctional CD8^+^ T cells that are specific to apoptotic epitopes could predict chronic infection in other acute (i.e., HBV or HIV) infections that develop viral persistence. The detection of these autoreactive CD8^+^ T cells may also be relevant in determining whether the contraction or the quality variation of the polyfunctional responses can be used as biomarkers to verify the protective effects of conventional or innovative antiviral therapies [62], [63].

## Materials and Methods

### Study population

The study cohort included 18HLA-A2^+^patients with acute HCV infection (5 women, 13 men, median age 34 years, range 22–57 years), according to the ethical guidelines of the 1975 Declaration of Helsinki and priori approval by the Ethics Committee of the Italian National Institute of Health: written informed consent was obtained from all patients. Diagnosis of acute HCV infection was based on (1) high levels of serum ALT; (2) seroconversion assessed by third generation enzyme linked immunosorbent assay, or anti-HCV positivity at the time of the diagnosis with an anti-HCV negative test in the previous 12 months; (3) presence of HCV-RNA in at least the first serum sample, and (4) sudden onset of liver disease symptoms. Alternative causes of acute hepatitis, such as other viral infection, autoimmunity, alcohol, drugs, and toxins were excluded. Patients with concomitant immunological disorders or with HIV coinfection were also excluded from the study.

### Cell preparations

PBMCs were isolated and T cell clones were generated as previously described [64]. CD8^+^ T cells were purified from PBMCs by positive selection coupled to magnetic beads (MiltenyiBiotec) as previously described [54]. Flow cytometry analysis demonstrated >99% CD8^+^ cells in the positively purified population and <5% in the CD8-depleted population. To purify antigen-specific type-17 CD8^+^ cells, PBMCs were stimulated with the relevant peptide plus anti-CD28 (4 µg/ml) (BD Pharmingen). Then, cells were stained with allophycocyanin (APC)-labeled anti-CCR6 (R&D System) and phycoerythin-cyanine 7 (PE-Cy7)-labeled anti-CCR4 (BD Pharmingen) and processed with FACSAria (Becton Dickinson) to sort CCR6^+^CCR4^+^ cells: >98% of these cells both produced IL-17 in response to the relevant peptide and were susceptible of staining with the relevant pentamers, as detected below. Spontaneous apoptosis of PBMCs from patients was determined by staining with Annexin-V (ApoAlert Apoptosis Kit, Clontech Laboratories Inc), propidium iodide (PI) (Sigma-Aldrich) and PE-Cy7-labeled anti-CD3 (BD Pharmingen) before and after 18 h incubation at 37°C. Immature (i)DCs were derived from peripheral monocytes that had been purified by positive selection with anti-CD14 mAb coupled to magnetic beads (MiltenyiBiotec). CD14^+^ cells were incubated for 5 days in RPMI 1640 medium containing 5% FCS, 2 mM glutamine, 1% nonessential amino acids, 1% sodium pyruvate, 50 µg/ml kanamycin (Gibco BRL), 50 ng/ml rGM-CFS (Novartis Pharma), and 1000 U/ml rIL-4 (gently provided by A. Lanzavecchia, Bellinzona, CH). Mature DCs were obtained by a 40-h stimulation of iDCs with soluble rCD40L molecules (Alexis Biochemicals, Alexis Corporation). The definition of monocyte-derived DCs was based on their surface phenotype profile by staining with anti-CD14, anti-CD86 (Caltag Laboratories), anti-CD1a, anti-CD1c, anti-CD11c, anti-CD32, anti-CD80 (BD PharMingen) mAbs, Annexin-V (ApoAlert Apoptosis Kit, Clontech Laboratories Inc), PI (Sigma-Aldrich), and the appropriate secondary labeled antibodies (BD PharMingen).

### ELISPOT assay *ex vivo*

Highly purified CD8^+^ T cells (1×10^5^) from PBMCs were stimulated for 4–6 h with nine independent pools of apoptotic peptides (**Table S1A–C**), eight independent pools of viral-peptides (genotype 1b), eight independent pools of viral-peptides (genotype 2c) [8], [25], or nine pools of overlapping peptides spanning the entire HCV genotype 3a (Chiron Mimotopes) (**Table S2A–E**), and irradiated autologous CD8-depleted PBMCs, used as APCs, and tested by an ELISPOT assay, as described [8]. Each peptide pool contained 5 µg/ml of each single peptide.

### Pentamer staining

PBMCs were incubated with APC-labeled–HLA-A*0201 pentamers (complexed to vimentin_78–87_ [LLQDSVDFSL], non-muscle myosin_478–486_ [QLFNHTMFI], or non-muscle myosin_741–749_ [VLMIKALEL] peptide) for apoptotic epitopes and APC-labeled–HLA-A*0201 pentamers (complexed to HCV-NS3_1073–1081_ [CINGVCWTV], HCV-NS3_1406–1415_ [KLVALGINAV] or HCV-Core_132–140_ [DLMGYIPAV] peptide) for viral epitopes (ProImmune Limited, Oxford, United Kingdom), in FACS buffer (PBS 1×, 2% human AB serum) for 10 min at 37°C, followed by washing and further incubation with APC-Cy7-labeled mAb to CD8 (BD Pharmingen, San Diego, CA), fluoresceinisothiocyanate (FITC)-labeled anti-PD-1 (BD Pharmingen), PE-labeled anti-CD127 (BD Pharmingen), FITC-labeled anti-CD45RO (Caltag Laboratories, Burlingame, CA) for 20 min at 4°C. Negative controls were obtained by staining cells with an irrelevant isotype-matched mAb. Cells were washed, acquired with a FACSCanto flow cytometer and FACSDiva analysis software (Becton Dickinson) or FlowJo software version 7.5.5 (Tree star, Inc. San Carlos, CA).

### Intracellular cytokine staining

PBMCs were stained with pentamers and mAb to CD8, and then stimulated with or without different concentrations of the corresponding uncomplexed peptide (ProImmune Limited) plus anti-CD28 mAb (4 µg/ml) (BD Pharmingen), or with PMA (50 ng/ml) plus ionomycin (1 µg/ml) (Sigma Aldrich, Milan, Italy), for 6 h at 37°C. At the 2nd h, 10 µg/ml Brefeldin A (Sigma-Aldrich) was added. Cells were washed, fixed and permeabilized using Cytofix/Cytoperm solution (BD Pharmingen) at 4°C for 20 min, re-washed with Perm Wash Buffer (BD Pharmingen), and intracellularly stained with different combinations of Alexa Fluor 488-labeled anti-IL17A (eBioscience San Diego, CA), PE-labeled anti-IFN-γ, PE-labeled anti-IL-2, FITC-labeled anti-IL-4 (BD Pharmingen), PE-labeled anti-RORC (eBioscience) or purified anti-T-bet (Santa Cruz Biotechnology Santa Cruz, California) for 30 min at 4°C. When stained with unlabeled specific antibody to detect T-bet, cells were washed and stained with the appropriate secondary FITC-labeled antibody. Cells were washed, acquired with a FACSCanto flow cytometer and FACSDiva analysis software (Becton Dickinson) or FlowJo software (Tree star).

### Cross-presentation of apoptotic cells

Cloned CD8^+^CD95^+^ T cells (10–100×10^6^) were incubated in the presence or absence of 14 µg/ml C3I (Z-DEVD-FMK), or a negative caspase control (K, Z-FA-FMK) (BD Pharmingen) for 1 h at 37°C in a 24-well plate. Then, cells were induced to apoptosis by incubation with 500 ng/ml anti-Fas (anti-CD95 mAb [clone CH11], Upstate Biotechnology) for at least 6 h. Apoptotic cells were determined by staining with Annexin-V (ApoAlert Apoptosis Kit, Clontech Laboratories Inc), PI (Sigma-Aldrich) and flow-cytometry analysis. PBMCs were double-stained with pentamers and mAb to CD8 and cultured with iDCs that had been pulsed or not with apoptotic cloned T cells. After 6–8 h, cells were tested for their capacity to produce IL-17 and IFN-γ by ICS as described above. Cells were washed, acquired with a FACSCanto flow cytometer and analyzed with FACSDiva analysis software (Becton Dickinson) or FlowJo software (Treestar).

### Pentamer staining decay (dissociation kinetics)

PBMCs were stained with saturating amounts of APC-labeled-HLA-A*0201 pentamers and APC-Cy7-labeled-CD8 (BD Pharmingen) for 45 min at room temperature [34]. Then, cells were washed three times with buffer (2% FCS, 0.01 sodium azide in PBS) and resuspended in 500 µl of buffer with saturanting amounts of mAb to HLA-A2 (BB7.2, ATCC). At various time points (0, 30 min, 1 h, 2 h and 3 h), an aliquot cells was washed and the fluorescence intensity was determined by flow cytometry analysis. Double staining using an anti-human TCRα/β (BD Pharmingen) and pentamers was performed in parallel to normalize pentamer fluorescence against the expressed TCR. The values were then normalized to percent of the total fluorescence at the initial time point and plotted on a logarithmic scale. *t*_1/2_ are determined by calculating the (ln2)/mean slope value of plots of the natural logarithm (ln) of the pentamer fluorescence normalized for the TCR fluorescence. The slope is equivalent to ln(Fa/Fb)/t, where Fa is the normalized fluorescence at the start of the interval, Fb is the normalized fluorescence at the end of the interval, and t is the length of the interval (minutes).

### T cell polarization

PBMCs were incubated for 10 days at 37°C with specific peptides (apoptotic or viral peptides), human rIL-6 (50 ng/ml), rIL-1β (10 ng/ml), rIL-23 (50 ng/ml) and rTGF-β (10 ng/ml) (R&D Systems) for the Th17 cell polarization. For the Th1 cell polarizing condition, PBMCs were antigen-stimulated in the presence of recombinant human rIL-12 (10 ng/ml) and rIFN-γ (100 U/ml) (R&D Systems). Recombinant IL-2 was added on day 4 of culture (50 U/ml). On day 10, cells were stained with surface antibodies, pentamers, anti-IL-17A, anti-IFN-γ, anti-IL-2 and anti-IL-4 mAbs. Cells were washed, acquired with a FACSCanto flow cytometer and analyzed with FACSDiva analysis software (Becton Dickinson) or FlowJo software (Tree star).

### Statistical analysis

All statistical analyses were performed with Prism 4 (GraphPad) software using nonparametric Spearman's correlation test, nonparametric Mann-Whitney U-test for unpaired data and Wilcoxon test for paired data. The differences were considered significant at P<0.05.

### Accession numbers

actin cytoplasmic 1 [ACTB] P60709

heterogeneous nuclear ribonucleoprotein [ROK] P61978

lamin B1 [LAM1] P20700

non muscle myosin heavy chain 9 [MYH9] P35579

vimentin [VIME] P08670

proteasome component C2 [PSA1] P25786

## Supporting Information

Figure S1 **Effector CD8^+^T cells specific to apoptotic or viral epitopes in patients with acute HCV infection.** Fresh CD8^+^T cells, purified from PBMCs of HLA-A2^+^patients undergoing chronic or self-limited HCV infection, were able to form IFN-γ spots promptly within 6 h of contact with autologous irradiated CD8-depleted PBMCs, as APCs, plus peptide *ex vivo*, as detected by an ELISPOT assay. Because of the limited number of PBMCs obtained from patients, freshly isolated CD8^+^T cells were tested against: 9 pools of apoptotic epitopes (see Table S1A–C), 8 pools of HLA-A2-binding peptides of HCV genotype 1c or genotype 2c, 9 pools of overlapping peptides spanning the entire HCV genotype 3a (see Table S2A–E). Each patient was studied with the viral peptides matched the own infecting genotype (see Table 1). Results are expressed as the number of peptide pools recognized in ELISPOT. Bars represent mean ± SD. P values have been calculated by comparing the number of recognized peptide pool in patients with chronic or resolved HCV infection at each time point.
(TIF)

Figure S2 **Polyfunctional CD8^+^ T_EM_ cells specific to apoptotic epitopes in patients with acute HCV infection.** Percentage of cells producing the indicated cytokines in CD8^+^pentamer^+^ cells in response to the indicated apoptotic epitopes (evaluated at the indicated time points by flow cytometry analyses) from patients with acute HCV infection experiencing chronic infection (filled symbols) or undergoing infection resolution (empty symbols). Circle symbols represent MYH9_478–485_ pentamer specificity, square symbols represent MYH9_741–749_ pentamer specificity, and triangle symbols represent VIME_78–87_ pentamer specificity. The horizontal dashed line delimits an arbitrary background, which is based on the values of 20 HLA-A2^+^ healthy individuals exhibiting <0.1% cytokine-producing cells in gated CD8^+^pentamer^+^ cells in each test.
(TIF)

Figure S3 **Polyfunctional CD8^+^ T_EM_ cells specific to viral epitopes in patients with acute HCV infection.** Percentage of cells producing the indicated cytokines in CD8^+^pentamer^+^ cells in response to the indicated viral epitopes (evaluated at the indicated time points by flow cytometry analyses) from patients with acute HCV infection experiencing chronic infection (filled symbols) or undergoing infection resolution (empty symbols). The horizontal dashed line delimits an arbitrary background, which is based on the values of 20 HLA-A2^+^ healthy individuals exhibiting <0.1% cytokine-producing cells in gated CD8^+^pentamer^+^ cells in each test. Circle symbols represent HCV-NS3_1073–1081_ pentamer specificity, square symbols represent HCV-NS3_1406–1415_ pentamer specificity, and triangle symbols represent HCV-Core_132–140_ pentamer specificity. NS = not significant.
(TIF)

Figure S4 **Mixed polyfunctional apoptotic epitope- or viral epitope-specific CD8+ T cell responses.** Number of patients exhibiting a wide repertoire of polyfunctional CD8^+^ T cells producing one or two cytokines in response to apoptotic (**A**) or viral (**B**) epitopes.
(TIF)

Figure S5 **Kinetics of fresh IFN-γ-producing CD8^+^ T cells specific to apoptotic epitopes.** PBMCs isolated from patients with acute HCV infection at the different time points indicated were stained with mAb to CD8 and pentamers complexed with the indicated apoptotic peptide. Cells were stimulated with the relevant soluble peptides plus anti-CD28 mAb for 6 h and then processed for the detection of IL-17 and IFN-γ by ICS assay with the relevant mAbs. Counterplot analyses are gated on CD8^+^pentamer^+^ cells and show percentages of cytokine-producing cells. The percentage of cells is reported in each quadrant.
(TIFF)

Figure S6 **No intrinsic defect of effector functions in CD8^+^ T cells from patients with acute HCV infection.** (**A**) One representative flow cytometry analysis in which PBMCs from patients with acute HCV infection were stained with mAb to CD8 and the indicated pentamer, stimulated with PMA and iono for 6 h, and processed for the detection of the indicated cytokines by ICS assay with the relevant mAbs. Counterplot analyses are gated on CD8^+^pentamer^+^ cells and show percentages of cytokine-producing cells. The percentage of cells is reported in each quadrant. (**B**) Percentage of cells producing the indicated cytokines in CD8^+^pentamer^+^ cells in response to PMA and iono (evaluated at the indicated time points by flow cytometry analyses) from patients with acute HCV infection experiencing chronic infection (filled symbols) or undergoing infection resolution (empty symbols).
(TIF)

Figure S7 **Naïve, central, and effector memory CD8^+^ T cells specific to apoptotic or viral epitopes.** (**A**) One representative flow cytometry analysis, the values of the total patients are shown in (B), in which PBMCs from a patient with acute HCV infection were stained with mAbs to CD8, CD45RO, and CD127 and with the pentamer complexed with the indicated apoptotic peptide. Counterplot analyses are gated on CD8^+^pentamer^+^ cells and show percentages of CD45RO^+^ and/or CD127^+^ cells. The percentage of cells is reported in each quadrant. (**B**) Percentages of CD45RO^+^CD127^−^, CD45RO^−^CD127^+^, or CD45RO^+^CD127^+^ cells in CD8^+^pentamer^+^ cells from aHCV patients experiencing chronic infection (filled circles) or infection resolution (empty circles). (**C**) One representative of six flow cytometry analyses in which PBMCs from a patient with acute HCV infection were stained with mAbs to CD8 and CD127 and with the pentamer complexed with the indicated peptide. Cells were stimulated with the same soluble peptide for 6 h and then processed for the detection of the indicated cytokines by ICS assay with the relevant mAbs.
(TIFF)

Figure S8 **PD-1 expression in CD8^+^ T cells specific to apoptotic or viral epitopes.** (**A,B**) Percentage of PD1^+^ cells in apoptotic (A) or viral (B) epitope-specific CD8^+^pentamer^+^ cells from patients with acute HCV infection experiencing chronic infection (filled circles) or undergoing infection resolution (empty circles). PBMCs, isolated at the different time points indicated, were stained with mAbs to CD8 and PD-1 and with pentamers complexed with the indicated peptides. In the panel A, circle symbols represent MYH9_478–485_ pentamer specificity, square symbols represent MYH9_741–749_ pentamer specificity, and triangle symbols represent VIME_78–87_ pentamer specificity. In the panel B, circle symbols represent HCV-NS3_1073–1081_ pentamer specificity, square symbols represent HCV-NS3_1406–1415_ pentamer specificity, and triangle symbols represent HCV-Core_132–140_ pentamer specificity. NS = not significant.
(TIFF)

Figure S9 **CD8^+^ T cells specific to apoptotic epitopes are accumulated in the liver from patients with long-term chronic HCV infection.** Representative flow cytometry analyses of PBMCs or intra-hepatic lymphocytes (IHLs) isolated from a liver biopsy of a HLA-A2^+^ patient with chronic HCV infection. Cells were double-stained with a mAb to CD8 and pentamers expressing the indicated peptide of MYH9. Analyses show the percentage of CD8^+^pentamer^+^ cells.
(TIFF)

Table S1 **HLA-A2 binding peptides derived from apoptotic cell-associated proteins appearing with their cleavage products in proteomic analysis.**
(PDF)

Table S2 **Peptides derived from HCV (Genotype 1b, Genotype 2c, or Genotype 3a) proteins used for the recognition by CD8^+^ T cells.** (**A,B**)HLA-A2 binding peptides derived from HCV Genotype 1b, or (**C,D**)HCV (Genotype 2c) proteins potentially recognized by HLA-A2-restricted HCV-specific CD8^+^ T cells. (**E**) Overlapping peptides spanning the entire HCV Genotype 3apotentially recognized by all HLA-restricted HCV-specific CD8^+^ T cells.
(PDF)
