# Supplementary figures and images for: Polyfunctional Type-1, -2, and -17 CD8+ T Cell Responses to Apoptotic Self-Antigens Correlate with the Chronic Evolution of Hepatitis C Virus Infection
Source: PLoS Pathog. 2012 Jun 21;8(6):e1002759. doi: 10.1371/journal.ppat.1002759 (PMC3380931; doi:10.1371/journal.ppat.1002759)

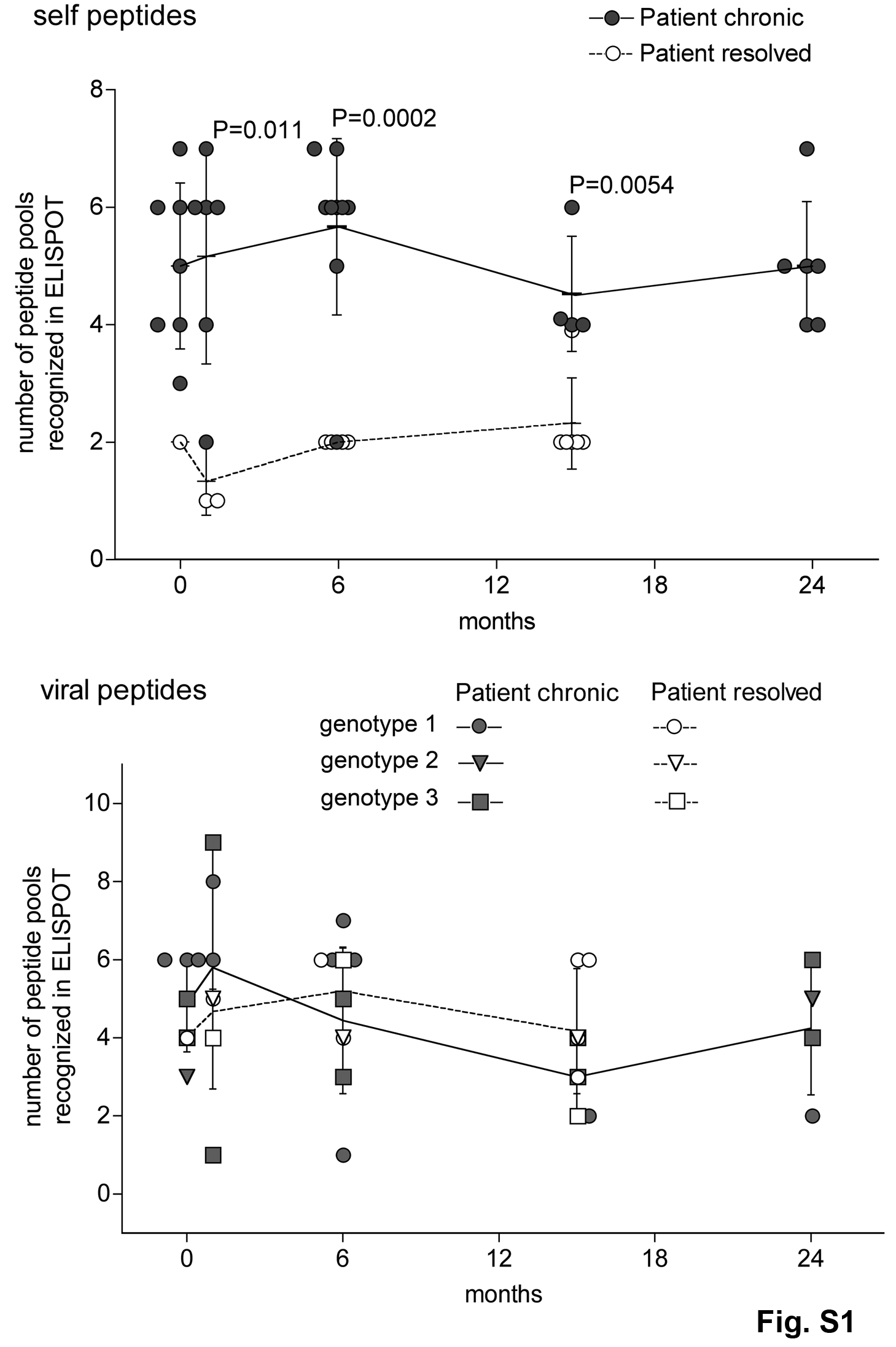

Supplement: Figure S1 — Effector CD8+T cells specific to apoptotic or viral epitopes in patients with acute HCV infection. Fresh CD8+T cells, purified from PBMCs of HLA-A2+patients undergoing chronic or self-limited HCV infection, were able to form IFN-γ spots promptly within 6 h of contact with autologous irradiated CD8-depleted PBMCs, as APCs, plus peptide ex vivo, as detected by an ELISPOT assay. Because of the limited number of PBMCs obtained from patients, freshly isolated CD8+T cells were tested against: 9 pools of apoptotic epitopes (see Table S1A–C), 8 pools of HLA-A2-binding peptides of HCV genotype 1c or genotype 2c, 9 pools of overlapping peptides spanning the entire HCV genotype 3a (see Table S2A–E). Each patient was studied with the viral peptides matched the own infecting genotype (see Table 1). Results are expressed as the number of peptide pools recognized in ELISPOT. Bars represent mean ± SD. P values have been calculated by comparing the number of recognized peptide pool in patients with chronic or resolved HCV infection at each time point. (TIF) [file ppat.1002759.s001.tif]

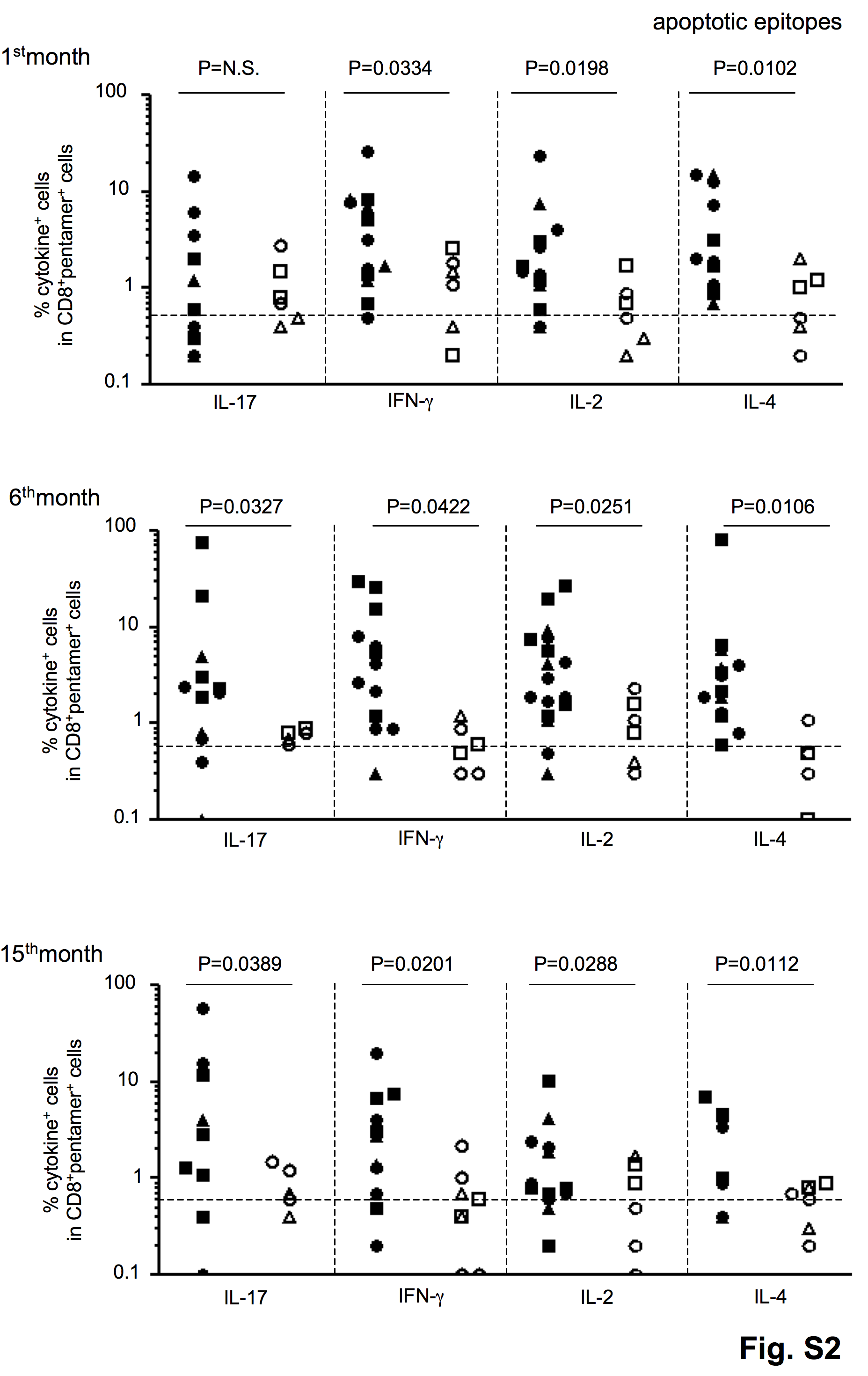

Supplement: Figure S2 — Polyfunctional CD8+ TEM cells specific to apoptotic epitopes in patients with acute HCV infection. Percentage of cells producing the indicated cytokines in CD8+pentamer+ cells in response to the indicated apoptotic epitopes (evaluated at the indicated time points by flow cytometry analyses) from patients with acute HCV infection experiencing chronic infection (filled symbols) or undergoing infection resolution (empty symbols). Circle symbols represent MYH9478–485 pentamer specificity, square symbols represent MYH9741–749 pentamer specificity, and triangle symbols represent VIME78–87 pentamer specificity. The horizontal dashed line delimits an arbitrary background, which is based on the values of 20 HLA-A2+ healthy individuals exhibiting <0.1% cytokine-producing cells in gated CD8+pentamer+ cells in each test. (TIF) [file ppat.1002759.s002.tif]

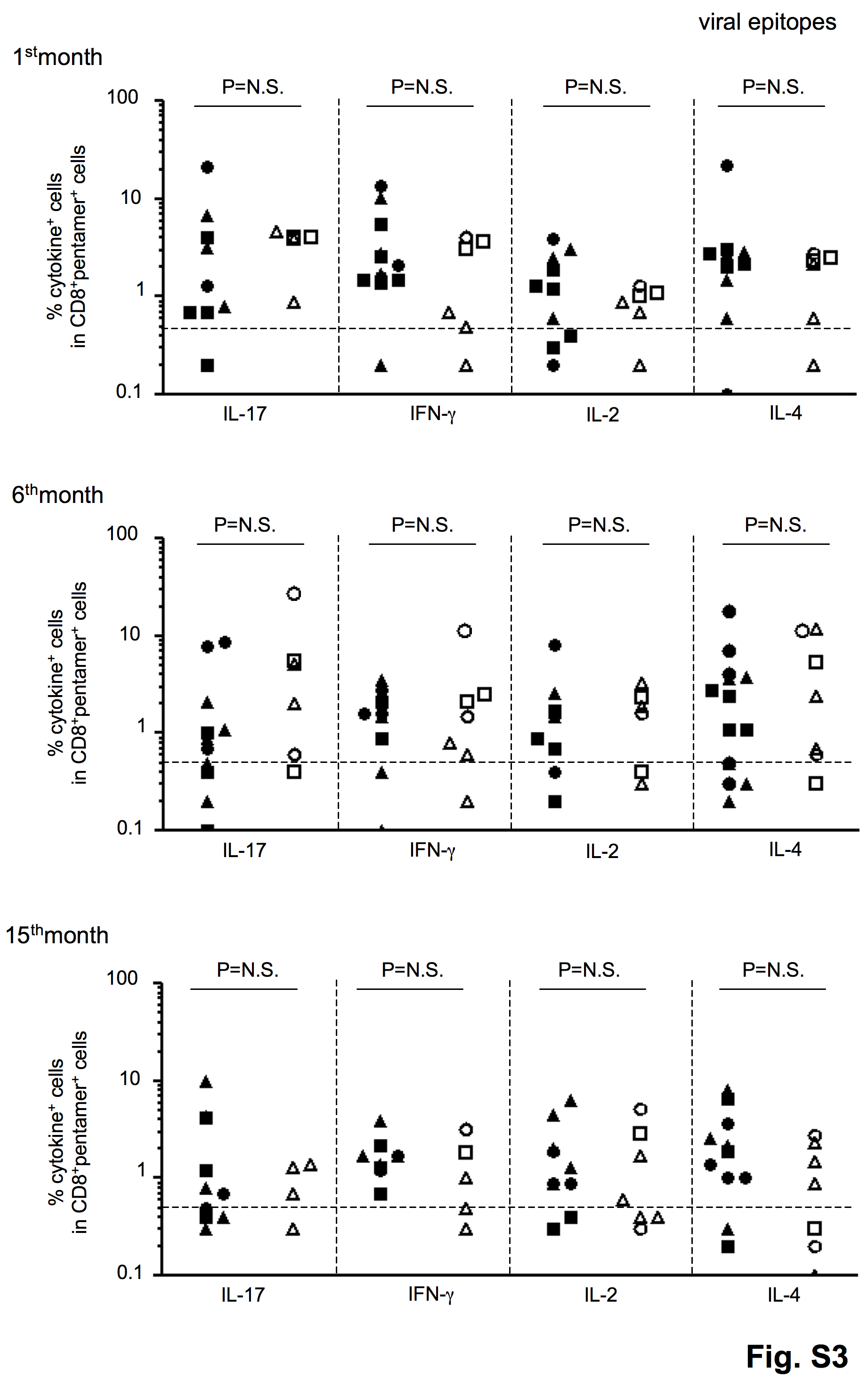

Supplement: Figure S3 — Polyfunctional CD8+ TEM cells specific to viral epitopes in patients with acute HCV infection. Percentage of cells producing the indicated cytokines in CD8+pentamer+ cells in response to the indicated viral epitopes (evaluated at the indicated time points by flow cytometry analyses) from patients with acute HCV infection experiencing chronic infection (filled symbols) or undergoing infection resolution (empty symbols). The horizontal dashed line delimits an arbitrary background, which is based on the values of 20 HLA-A2+ healthy individuals exhibiting <0.1% cytokine-producing cells in gated CD8+pentamer+ cells in each test. Circle symbols represent HCV-NS31073–1081 pentamer specificity, square symbols represent HCV-NS31406–1415 pentamer specificity, and triangle symbols represent HCV-Core132–140 pentamer specificity. NS = not significant. (TIF) [file ppat.1002759.s003.tif]

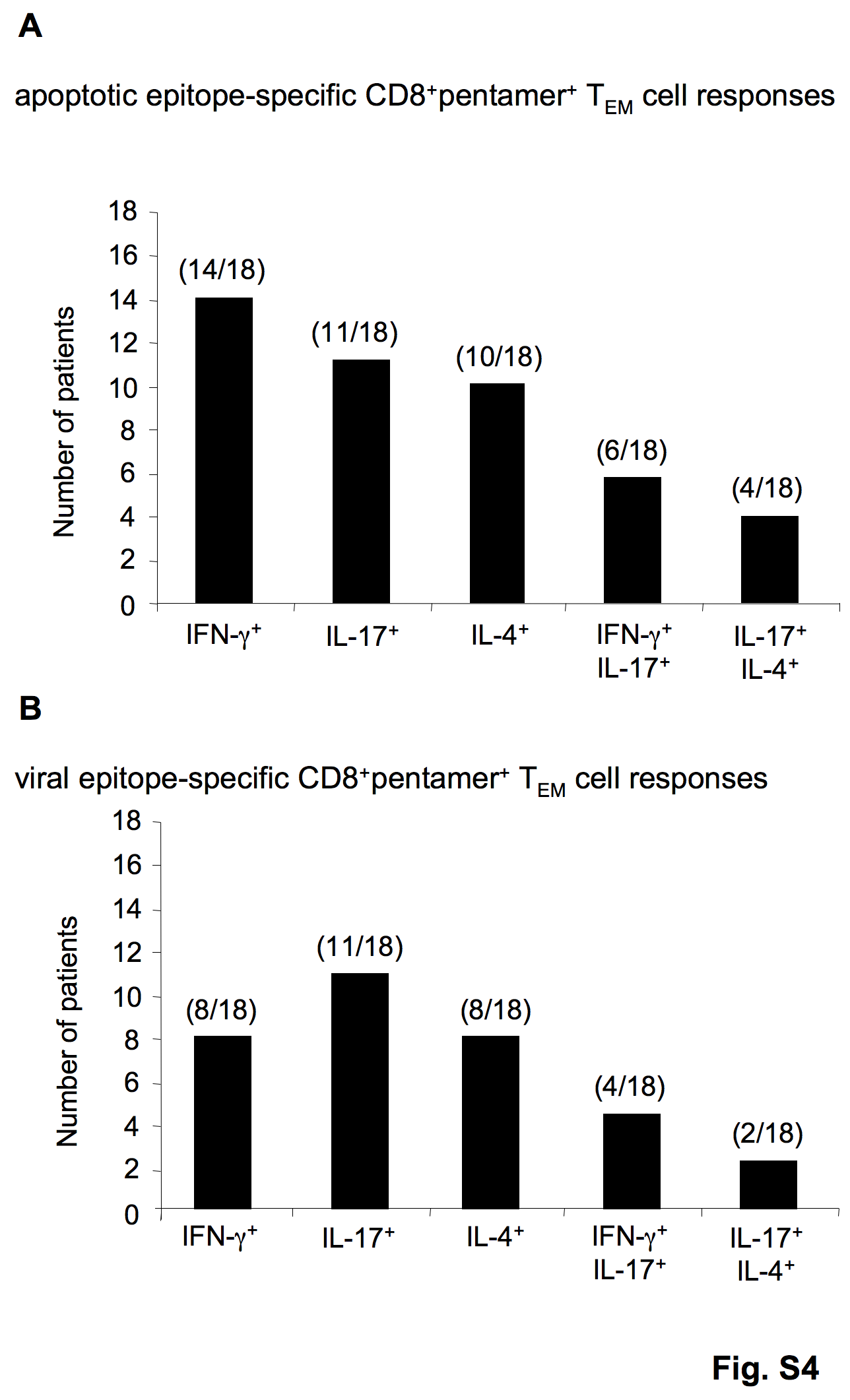

Supplement: Figure S4 — Mixed polyfunctional apoptotic epitope- or viral epitope-specific CD8+ T cell responses. Number of patients exhibiting a wide repertoire of polyfunctional CD8+ T cells producing one or two cytokines in response to apoptotic (A) or viral (B) epitopes. (TIF) [file ppat.1002759.s004.tif]

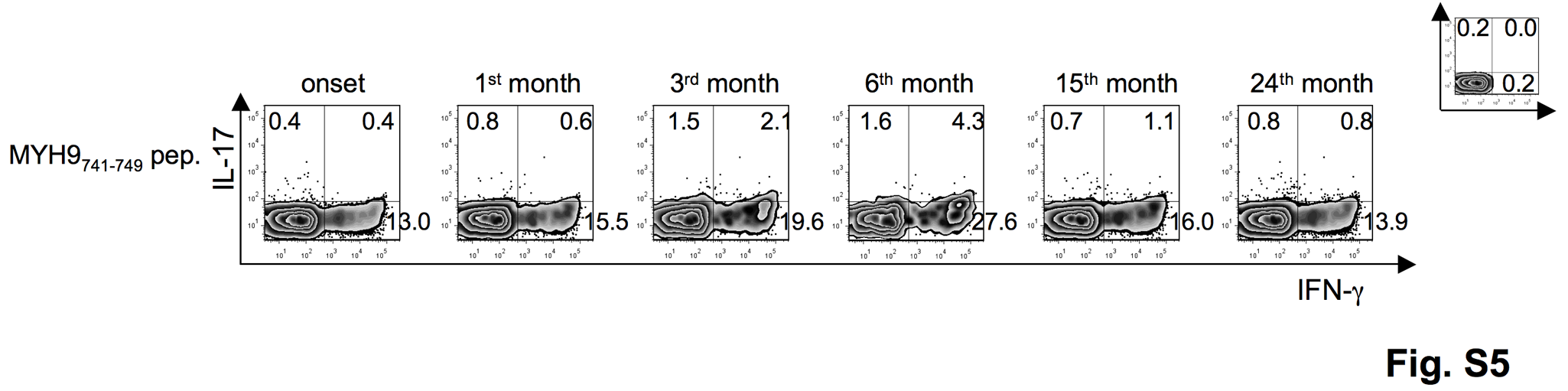

Supplement: Figure S5 — Kinetics of fresh IFN-γ-producing CD8+ T cells specific to apoptotic epitopes. PBMCs isolated from patients with acute HCV infection at the different time points indicated were stained with mAb to CD8 and pentamers complexed with the indicated apoptotic peptide. Cells were stimulated with the relevant soluble peptides plus anti-CD28 mAb for 6 h and then processed for the detection of IL-17 and IFN-γ by ICS assay with the relevant mAbs. Counterplot analyses are gated on CD8+pentamer+ cells and show percentages of cytokine-producing cells. The percentage of cells is reported in each quadrant. (TIFF) [file ppat.1002759.s005.tif]

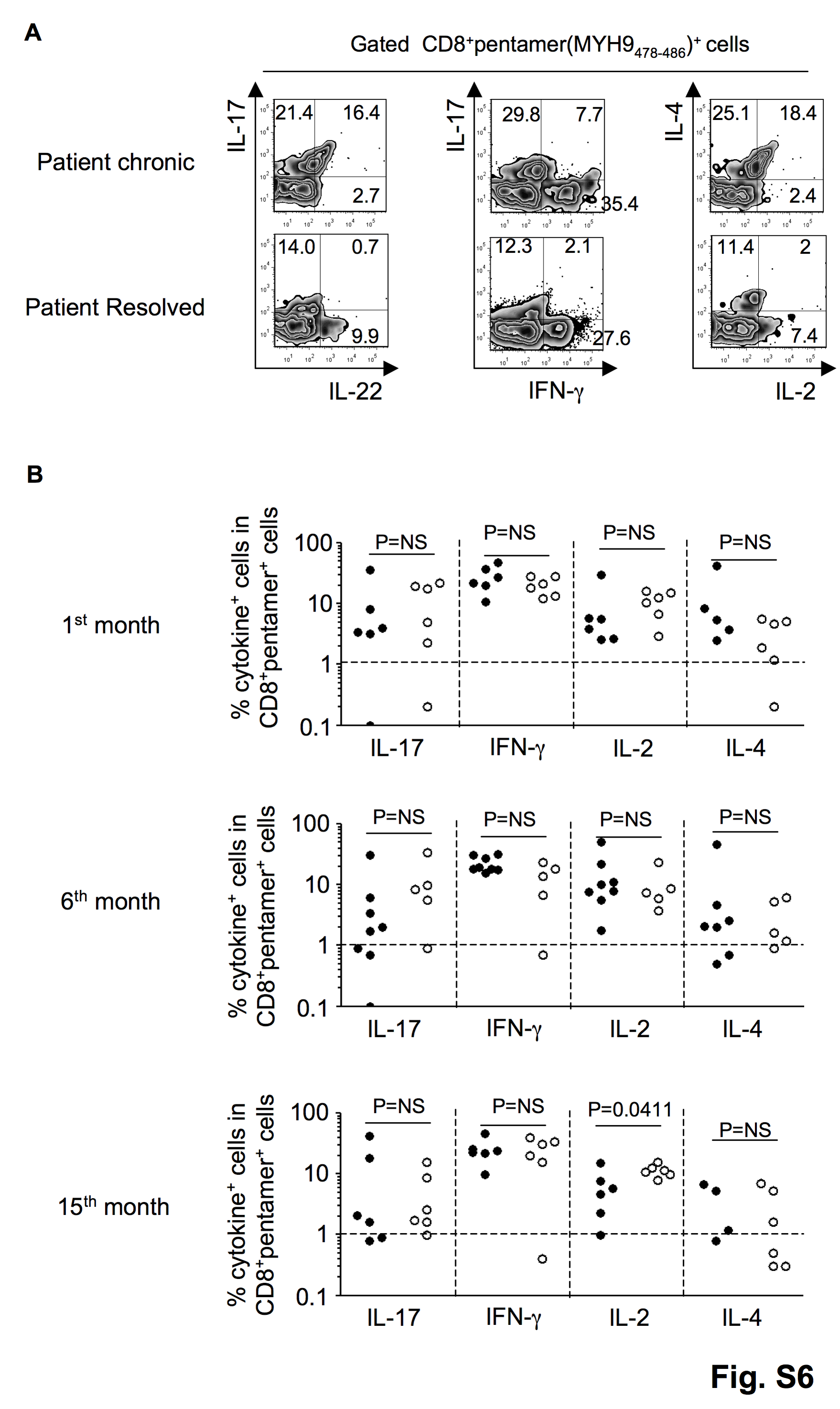

Supplement: Figure S6 — No intrinsic defect of effector functions in CD8+ T cells from patients with acute HCV infection. (A) One representative flow cytometry analysis in which PBMCs from patients with acute HCV infection were stained with mAb to CD8 and the indicated pentamer, stimulated with PMA and iono for 6 h, and processed for the detection of the indicated cytokines by ICS assay with the relevant mAbs. Counterplot analyses are gated on CD8+pentamer+ cells and show percentages of cytokine-producing cells. The percentage of cells is reported in each quadrant. (B) Percentage of cells producing the indicated cytokines in CD8+pentamer+ cells in response to PMA and iono (evaluated at the indicated time points by flow cytometry analyses) from patients with acute HCV infection experiencing chronic infection (filled symbols) or undergoing infection resolution (empty symbols). (TIF) [file ppat.1002759.s006.tif]

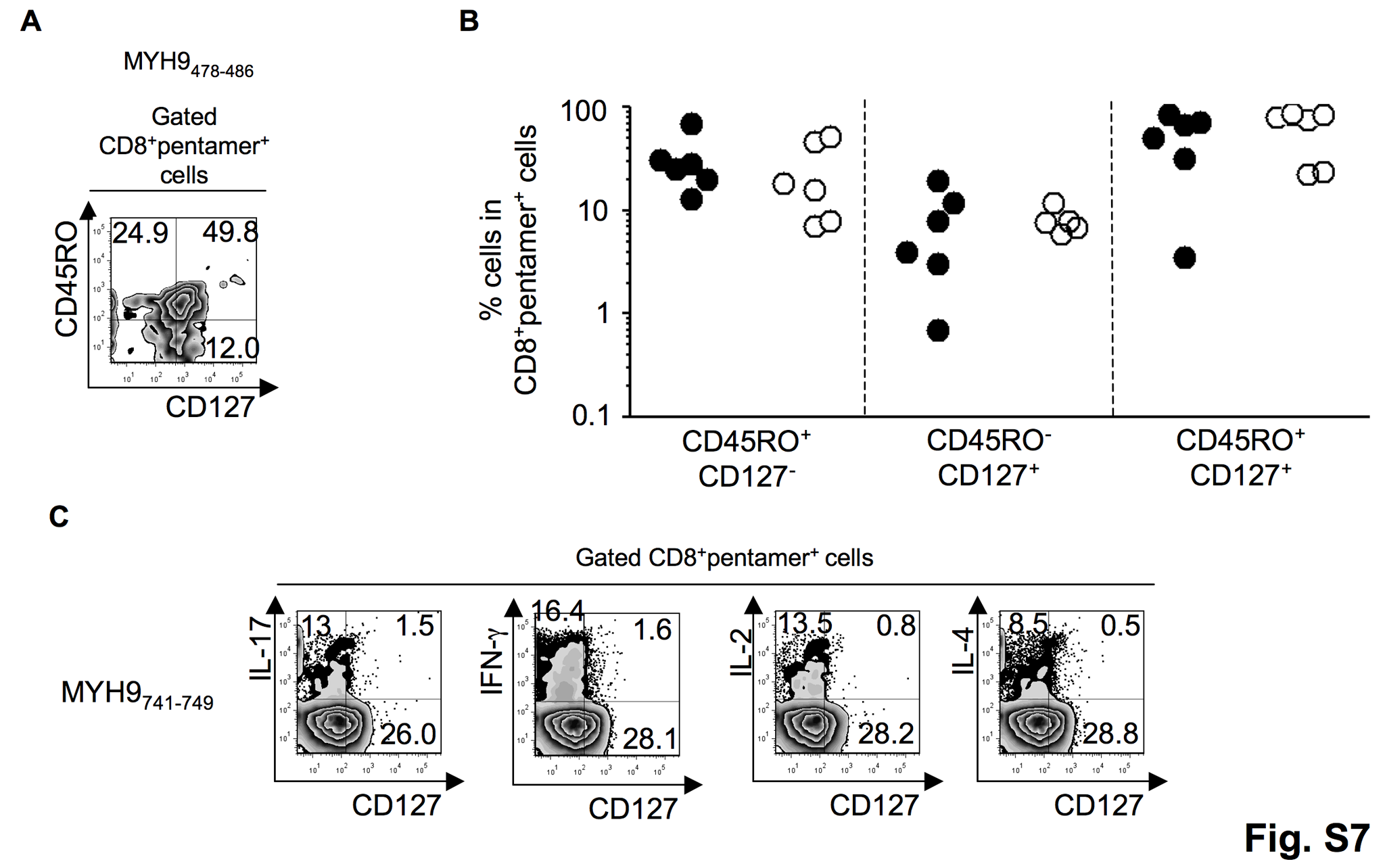

Supplement: Figure S7 — Naïve, central, and effector memory CD8+ T cells specific to apoptotic or viral epitopes. (A) One representative flow cytometry analysis, the values of the total patients are shown in (B), in which PBMCs from a patient with acute HCV infection were stained with mAbs to CD8, CD45RO, and CD127 and with the pentamer complexed with the indicated apoptotic peptide. Counterplot analyses are gated on CD8+pentamer+ cells and show percentages of CD45RO+ and/or CD127+ cells. The percentage of cells is reported in each quadrant. (B) Percentages of CD45RO+CD127−, CD45RO−CD127+, or CD45RO+CD127+ cells in CD8+pentamer+ cells from aHCV patients experiencing chronic infection (filled circles) or infection resolution (empty circles). (C) One representative of six flow cytometry analyses in which PBMCs from a patient with acute HCV infection were stained with mAbs to CD8 and CD127 and with the pentamer complexed with the indicated peptide. Cells were stimulated with the same soluble peptide for 6 h and then processed for the detection of the indicated cytokines by ICS assay with the relevant mAbs. (TIFF) [file ppat.1002759.s007.tif]

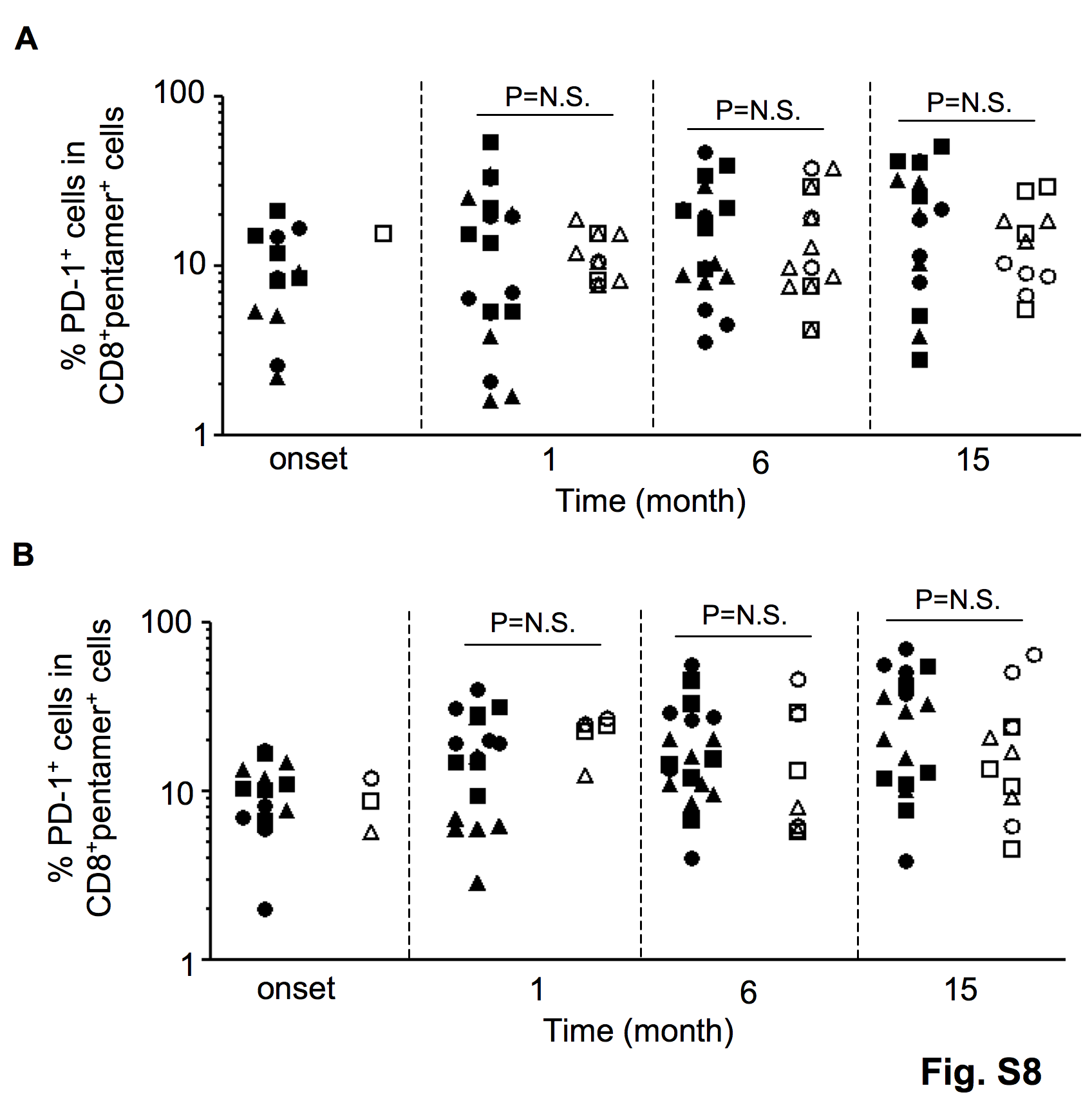

Supplement: Figure S8 — PD-1 expression in CD8+ T cells specific to apoptotic or viral epitopes. (A,B) Percentage of PD1+ cells in apoptotic (A) or viral (B) epitope-specific CD8+pentamer+ cells from patients with acute HCV infection experiencing chronic infection (filled circles) or undergoing infection resolution (empty circles). PBMCs, isolated at the different time points indicated, were stained with mAbs to CD8 and PD-1 and with pentamers complexed with the indicated peptides. In the panel A, circle symbols represent MYH9478–485 pentamer specificity, square symbols represent MYH9741–749 pentamer specificity, and triangle symbols represent VIME78–87 pentamer specificity. In the panel B, circle symbols represent HCV-NS31073–1081 pentamer specificity, square symbols represent HCV-NS31406–1415 pentamer specificity, and triangle symbols represent HCV-Core132–140 pentamer specificity. NS = not significant. (TIFF) [file ppat.1002759.s008.tif]

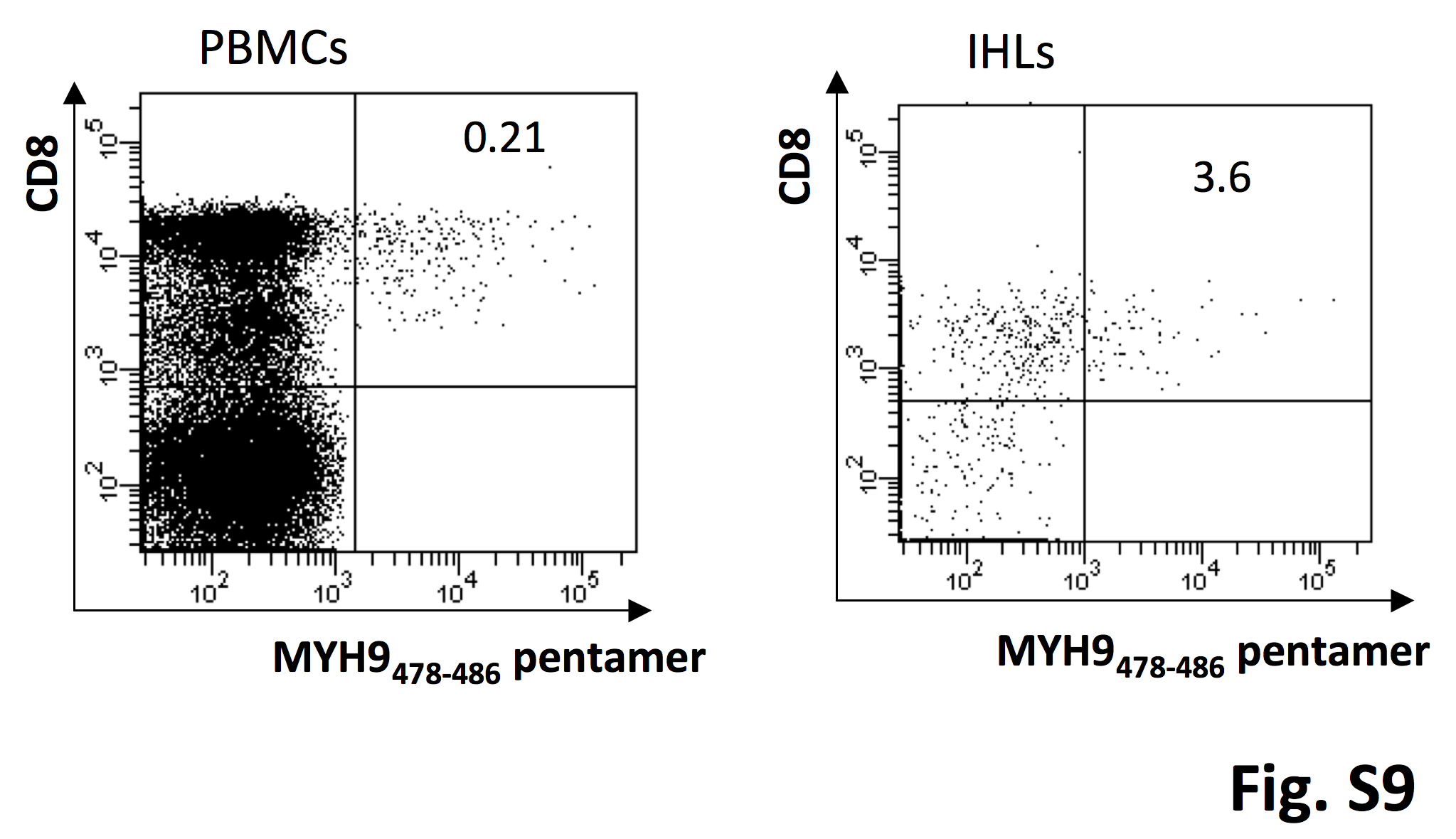

Supplement: Figure S9 — CD8+ T cells specific to apoptotic epitopes are accumulated in the liver from patients with long-term chronic HCV infection. Representative flow cytometry analyses of PBMCs or intra-hepatic lymphocytes (IHLs) isolated from a liver biopsy of a HLA-A2+ patient with chronic HCV infection. Cells were double-stained with a mAb to CD8 and pentamers expressing the indicated peptide of MYH9. Analyses show the percentage of CD8+pentamer+ cells. (TIFF) [file ppat.1002759.s009.tif]
