# Supplementary material for: Polyfunctional Type-1, -2, and -17 CD8+ T Cell Responses to Apoptotic Self-Antigens Correlate with the Chronic Evolution of Hepatitis C Virus Infection
Source: PLoS Pathog. 2012 Jun 21;8(6):e1002759. doi: 10.1371/journal.ppat.1002759 (PMC3380931; doi:10.1371/journal.ppat.1002759)
Supplement: Table S1 — HLA-A2 binding peptides derived from apoptotic cell-associated proteins appearing with their cleavage products in proteomic analysis. (PDF) [file ppat.1002759.s010.pdf]

Table S1A: HLA-A2 binding peptides derived from apoptotic cell-associated proteins appearing with their cleavage products in proteomic analysis.

						Binding capacity (IC50§ nM)					Alleles
Pool	Organism	Protein	1st Pos°	Sequence	Length	A*0201	A*0202	A*0203	A*0206	A*6802	bound
1	Human	ACTB"	131	AMYVAIQAV	9	2.8	1.5	1.6	7.1	190	5
	Human	ACTB	319	ALAPSTMKI	9	4.3	2.9	2.5	40	5.5	5
	Human	ACTB	266	FLGMESCGI	9	2.7	71	11	16	3979	4
	Human	ACTB	312	RMQKEITAL	9	14	0.29	6.5	28	6667	4
	Human	ACTB	348	SLSTFQQMWI	10	5.6	4.5	7.7	791	299	4
	Human	ACTB	46	GMGQKDSYV	9	196	17	156	13547	17632	3
2	Human	ROK^	154	SLAGGIIGV	9	0.56	1.0	1.1	0.14	49	5
	Human	ROK	67	ALRTDYNASV	10	8.9	5.0	11	349	1354	4
	Human	ROK	193	VLIGGKPDRV	10	14	8.5	12	1282	7417	3
	Human	ROK	209	ILDLISESPI	10	360	65	1272	2598	3083	2
	Human	ROK	122	QLPLESDAV	9	246	601	1270	925	1999	1
3	Human	LAM1*	496	TIWAANAGV	9	8.9	263	308	22	20	5
	Human	LAM1	41	RLAVYIDKV	9	0.16	0.84	0.18	14	1004	4
	Human	LAM1	301	SLSSQLSNL	9	1.6	0.57	2.4	104	1377	4
	Human	LAM1	361	QLLDVKLAL	9	0.060	98	288	4.0	5848	4
	Human	LAM1	291	ELMESRMRI	9	246	1226	284	25188	45	3
	Human	LAM1	355	QLNDYEQLL	9	2.0	0.23	18	1493	4808	3
	Human	LAM1	388	KLSPSPSSRV	10	50	18	45	563	20979	3
	Human	LAM1	488	VLKAGQTVTI	10	263	37	23	4060	15429	3
	Human	LAM1	378	KLLEGEEERL	10	30	29	3395	1944	20515	2
4	Human	MYH9**	9	YLYVDKNFI	9	9.3	0.85	4.8	45	217	5
	Human	MYH9	108	GLIYTYSGL	9	22	11	10	197	58	5
	Human	MYH9	111	YTYSGLFCV	9	0.12	2.1	3.0	0.84	3.2	5
	Human	MYH9	145	EMPPHIYAI	9	323	67	110	85	1.8	5
	Human	MYH9	186	KVIQYLAYV	9	0.67	0.17	1.2	0.21	1.8	5
	Human	MYH9	478	QLFNHTMFI	9	1.4	0.71	3.2	14	14	5
	Human	MYH9	584	WLMKNMDPL	9	9.7	0.36	14	6.0	1.9	5
	Human	MYH9	653	QLAKLMATL	9	5.4	1.4	2.9	26	18	5
	Human	MYH9	111	YTYSGLFCVV	10	0.21	0.34	0.12	4.2	0.4	5

°=1st amino acid position; §=IC50 indicates binding affinity and it is expressed as 50% inhibitory nanomolar concentration, a dash ("-") indicates an IC50 >50000 nM; peptides with IC50 <50nM are classified as high affinity peptides, peptides with IC50>50 and <500nM are classified as intermediate affinity peptides, peptides with IC50>500nM are classified as low affinity peptides; "=actin cytoplasmatic 1; ^=eterogeneous nuclear ribonucleoprotein K; *=lamin B1; **=non muscle myosin.

Table S1B: HLA-A2 binding peptides derived from apoptotic cell-associated proteins appearing with their cleavage products in proteomic analysis.

Pool	Organism	Protein	1st Pos°	Sequence	Length	Binding capacity (IC50§ nM)					Alleles
						A*0201	A*0202	A*0203	A*0206	A*6802	bound
5	Human	MYH9**	424	RMFRWLVLRI	10	1.1	5.0	10	71	398	5
	Human	MYH9	478	QLFNHTMFIL	10	0.97	0.42	8.2	49	82	5
	Human	MYH9	302	FLSNGHVTI	9	0.11	1.0	3.8	11	3050	4
	Human	MYH9	338	GLLRVISGV	9	0.21	3.0	3.1	4.3	8823	4
	Human	MYH9	412	FAIEALAKA	9	26	1.7	4.0	0.43	-	4
	Human	MYH9	450	ILDIAGFEI	9	5.5	50	257	21	3064	4
	Human	MYH9	733	FMDGKQACV	9	0.18	7.8	33	6.6	10345	4
	Human	MYH9	741	VLMIKALEL	9	0.15	15	38	78	870	4
	Human	MYH9	1277	KLQVELDNV	9	4.1	8.8	30	9.2	-	4
	Human	MYH9	1843	KLKDVLLQV	9	3.3	2.5	6.7	0.20	18519	4
	Human	MYH9	279	YLLSGAGEHL	10	4.9	6.3	175	11	-	4
	Human	MYH9	733	FMDGKQACVL	10	21	27	158	683	374	4
6	Human	MYH9	1920	KLRRGDLPFV	10	2.9	15	9.1	5.5	-	4
	Human	MYH9	210	QLLQANPIL	9	4.5	183	1507	19	-	3
	Human	MYH9	847	MMAKEEELV	9	9.1	1.2	276	1070	5639	3
	Human	MYH9	877	QLMAEKLQL	9	8.2	7.9	205	657	4360	3
	Human	MYH9	1726	RLEARIAQL	9	433	7.7	15	2292	46881	3
	Human	MYH9	1793	KLQEMEGTV	9	23	99	48	867	19355	3
	Human	MYH9	660	TLRNTNPNFV	10	21	107	40	-	593	3
	Human	MYH9	688	VLDQLRCNGV	10	6.6	186	61	868	-	3
	Human	MYH9	752	NLYRIGQSKV	10	322	87	76	17848	1024	3
	Human	MYH9	248	YIVGANIET	9	422	622	9009	81	626	2
	Human	MYH9	1540	QLEELEDEL	9	404	176	-	28866	4718	2
	Human	MYH9	161	MMQDREDQSI	10	2.1	25	542	1245	4005	2
	Human	MYH9	821	KLRNWQWWRL	10	56	54	1037	5442	-	2
	Human	MYH9	846	EMMAKEEELV	10	33	9.6	958	2456	2809	2

°=1st amino acid position; §=IC50 indicates binding affinity and it is expressed as 50% inhibitory nanomolar concentration, a dash ("-") indicates an IC50 >50000 nM; peptides with IC50 <50nM are classified as high affinity peptides, peptides with IC50>50 and <500nM are classified as intermediate affinity peptides, peptides with IC50>500nM are classified as low affinity peptides; **=non muscle myosin.

Table S1C: HLA-A2 binding peptides derived from apoptotic cell-associated proteins appearing with their cleavage products in proteomic analysis.

Pool	Organism	Protein	1st Pos°	Sequence	Length	Binding capacity (IC50§ nM)					Alleles
						A*0201	A*0202	A*0203	A*0206	A*6802	bound
7	Human	VIME%	176	NLAEDIMRL	9	1.0	0.31	4.2	136	83	5
	Human	VIME	50	SLYASSPGGV	10	2.2	4.3	2.4	461	452	5
	Human	VIME	68	RLRSSVPGV	9	3.3	27	1.6	30	9901	4
	Human	VIME	129	ILLAELEQL	9	35	6.5	231	7.5	-	4
	Human	VIME	225	SLQEEIAFL	9	0.99	0.37	19	44	4777	4
	Human	VIME	78	LLQDSVDFSL	10	3.6	1.1	149	9.2	1931	4
	Human	VIME	79	LQDSVDFSL	9	5.9	47	10155	2.3	774	3
	Human	VIME	419	SLNLRETNL	9	402	1.4	22	16458	-	3
	Human	VIME	122	FLEQQNKILL	10	20	0.43	63	828	10669	3
	Human	VIME	370	NMKEEMARHL	10	260	433	703	10578	9346	2
8	Human	PSA1***	179	FMECNLNEL	9	596	27	55	15067	14822	2
	Human	PSA1	183	NLNELVKHGL	10	145	12	11	4834	3618	3
	Human	PSA1	175	HMSEFMECNL	10	59	11	70	109	35360	4
	Human	PSA1	63	ILHVDNHIGI	10	-	-	16720	-	-	0
	Human	PSA1	37	GLKSKTHAV	9	-	4552	924	-	19982	0
	Human	PSA1	110	SLIGSKTQI	9	-	15913	511	34100	17135	0
	Human	PSA1	179	FMECNLNELV	10	12715	194	1	-	-	2
	Human	PSA1	48	ALKRAQSEL	9	-	-	18952	-	29040	0
	Human	PSA1	76	GLTADARLL	9	163	4.7	1	7.8	12	5
	Human	PSA1	102	PLPVSRLVSL	10	4.5	1.2	0.21	40	1659	4
	Human	PSA1	204	DLTTKNVSI	9	465	3.6	54	4278	7386	3
	Human	PSA1	45	ELNGKNIEDV	10	104	1.2	0.21	1776	14417	3
	Human	PSA1	55	ELAAHQKKI	9	38	2	1.1	100	9790	4
9	Human	PSA1	186	ELVKHGLRAL	10	296	15	1.6	8411	-	3
	Human	PSA1	37	GLKSKTHAVL	10	2366	116	24	10609	21672	2
	Human	PSA1	191	GLRALRETL	9	41760	45	23	-	-	2
	Human	PSA1	55	ELAAHQKKIL	10	8856	146	362	16700	-	2
	Human	PSA1	97	FVFDRPLPV	9	-	8417	2175	9732	44603	0

°=1st amino acid position; §=IC50 indicates binding affinity and it is expressed as 50% inhibitory nanomolar concentration, a dash ("-") indicates an IC50 >50000 nM; peptides with IC50 <50nM are classified as high affinity peptides, peptides with IC50>50 and <500nM are classified as intermediate affinity peptides, peptides with IC50>500nM are classified as low affinity peptides; %=vimentin; ***PSA1= Proteasome component C2.
