# Supplementary material for: Polyfunctional Type-1, -2, and -17 CD8+ T Cell Responses to Apoptotic Self-Antigens Correlate with the Chronic Evolution of Hepatitis C Virus Infection
Source: PLoS Pathog. 2012 Jun 21;8(6):e1002759. doi: 10.1371/journal.ppat.1002759 (PMC3380931; doi:10.1371/journal.ppat.1002759)
Supplement: Table S2 — Peptides derived from HCV (Genotype 1b, Genotype 2c, or Genotype 3a) proteins used for the recognition by CD8+ T cells. (A,B)HLA-A2 binding peptides derived from HCV Genotype 1b, or (C,D)HCV (Genotype 2c) proteins potentially recognized by HLA-A2-restricted HCV-specific CD8+ T cells. (E) Overlapping peptides spanning the entire HCV Genotype 3apotentially recognized by all HLA-restricted HCV-specific CD8+ T cells. (PDF) [file ppat.1002759.s011.pdf]

Table S2A. HLA-A2 binding peptides derived from Genotype 1b HCV Core, E1, E2, NS2, and NS3 proteins potentially recognized by HLA-A2-restricted virus-specific CD8^{+} T cells.

Pool	Organism	Protein	1st Pos°	Sequence	Length
1	Human	HCV Core protein	35	YLLPRRGPRL	10
	Human	HCV Core protein	132	DLMGYIPLV	9
	Human	HCV Core protein	177	FLLALLSCL	9
	Human	HCV Core protein	177	FLLALLSCLT	10
	Human	HCV Core protein	180	ALLSCLTTPA	10
	Human	HCV Core protein	181	LLSCLTTPA	9
2	Human	E_1 protein	322a	MMMNWSPTT	9
	Human	E_1 protein	322b	MMMNWSPTTA	10
	Human	E_1 protein	362	YSMAGNWAKV	10
	Human	E_1 protein	371	VLIVMLLFA	9
	Human	E_1 protein	372	LIVMLLFAGW	10
	Human	E_2 protein	614	RLWHYPCTV	9
	Human	E_2 protein	665	LLLSTTEWQV	10
	Human	E_2 protein	666	LLSTTEWQV	9
3	Human	E_2 protein	701	YLYGIGSAV	9
	Human	E_2 protein	701	YLYGIGSAVV	10
	Human	E_2 protein	718	YVLLLFLLLA	10
	Human	E_2 protein	719	VLLLFLLLA	9
	Human	E_2 protein	723	FLLLADARV	9
	Human	E_2 protein	736	WMMLLIAQA	9
	Human	E_2 protein	738	MLLIAQAEA	9
4	Human	NS2	838	FLARLIWWL	9
	Human	NS2	875	LLMCAVHPEL	10
	Human	NS2	890	KLLIAILGPL	10
	Human	NS2	891	LLIAILGPL	9
	Human	NS2	901	VLQAGITRV	9
	Human	NS2	917	GLIHACMLV	9
	Human	NS2	940	KLGALTGYI	10
	Human	NS2	948	YIYNHLTPL	9
5	Human	NS3	1038	GLLGCIITSL	10
	Human	NS3	1039	LLGCIITSL	9
	Human	NS3	1069	FLATCVNGV	9
	Human	NS3	1406	KLSGLGINAV	10
	Human	NS3	1542	YLNTPGLPV	9
	Human	NS3	1585	YLVAYQATV	9
	Human	NS3	1606	QMWKCLIRL	9

Table S2B. HLA-A2 binding peptides derived from Genotype 1b HCV NS4 and NS5 proteins potentially recognized by HLA-A2-restricted virus-specific CD8^{+} T cells.

Pool	Organism	Protein	1st Pos°	Sequence	Length
6	Human	NS4A	1661	VLVGGVLAAL	10
	Human	NS4B	1764	HMWNFISGI	9
	Human	NS4B	1768	FISGIQYLA	9
	Human	NS4B	1789	SLMAFTASI	9
	Human	NS4B	1807	LLFNILGGWV	10
	Human	NS4B	1851	ILAGYGAGV	9
7	Human	NS5A	1998	KLLPQLPGV	9
	Human	NS5A	2077	RLIVFPDLGV	10
	Human	NS5A	2093	ALYDVVSTL	9
	Human	NS5B	2726	KLQDCTMLV	9
	Human	NS5B	3732	MLVNGDDLVV	10
	Human	NS5B	2827	WLGNIIMYA	9
	Human	NS5B	2842	MILMTHFFSI	10
8	Human	NS5B	2843	ILMTHFFSI	9
	Human	NS5B	2843	ILMTHFFSIL	10
	Human	NS5B	2861	ALDCQIYGA	9
	Human	NS5B	2884	RLHGLSAFSL	10
	Human	NS5B	2991	FMLCLLLLS	9
	Human	NS5B	299	FMLCLLLLSV	10
	Human	NS5B	2992	MLCLLLLSV	9
	Human	NS5B	2995	LLLLSVGVGI	10
	Human	NS5B	2997	LLSVGVGIYL	10

Table S2C. HLA-A2 binding peptides derived from Genotype 2c HCV Core, E1, E2, p27, NS2, and NS3 proteins potentially recognized by HLA-A2-restricted virus-specific CD8⁺ T cells.

Pool	Organism	Protein	1th Pos°	Sequence	Length
1	Human	HCV Core protein	177	FLLALLSCI	9
	Human	HCV Core protein	178	LLALLSCISV	10
	Human	HCV Core protein	180	ALLSCISVPV	10
	Human	HCV Core protein	181	LLSCISVPV	9
2	Human	E_1 protein	283	ALMIAAQVVV	9
	Human	E_1 protein	332	MLLAYLVRI	10
	Human	E_1 protein	355	VMFGLAYFSM	10
	Human	E_1 protein	362	FSMQGAWAKV	9
	Human	E_2 protein	397	HLFTSMFSL	10
	Human	E_2 protein	437	FLAALFYTS	9
	Human	E_2 protein	705	YLYGLSPAI	10
3	Human	p27	768	GLLYFILFFV	10
	Human	p27	769	LLYFILFFV	9
	Human	p27	769	LLYFILFFVA	10
	Human	p27	792	YTLLGCWSFV	10
	Human	p27	793	TLLGCWSFV	9
	Human	p27	793	TLLGCWSFVL	10
	Human	p27	803	LLMALPHQA	9
4	Human	NS2	826	LLIAITAF	9
	Human	NS2	841	ILLSRCLWWT	10
	Human	NS2	846	CLWWTCYMLV	10
	Human	NS2	851	YMLVLAEALI	10
	Human	NS2	859	LIQDWIPPL	9
	Human	NS2	880	AMTMFYPGV	9
	Human	NS2	1001	ALLRMCALV	9
	Human	NS2	1027	RLLAPITAYA	10
	Human	NS2	1028	LLAPITAYA	9
5	Human	NS3	1073	FLGTSISGV	9
	Human	NS3	1076	TSISGVLWTV	10
	Human	NS3	1077	SISGVLWTV	9
	Human	NS3	1081	VLWTVFHGA	9
	Human	NS3	1135	YLVTRNADV	9
	Human	NS3	1610	VMWKCLIRL	9

Table S2D. HLA-A2 binding peptides derived from Genotype 2c HCV NS4 and NS5 proteins potentially recognized by HLA-A2-restricted virus-specific CD8+ T cells.

Pool	Organism	Protein	1th Pos°	Sequence	Length
6	Human	NS4A	1665	VLAGGVLAAV	10
	Human	NS4B	1793	SMMSFSAAL	9
	Human	NS4B	1855	VLAGYGAGI	9
	Human	NS4B	1871	KIMSGEKPSV	10
7	Human	NS5A	2002	KLFPRLPGI	9
	Human	NS5A	2144	FMRDEVSFSV	10
	Human	NS5A	2254	VLMVDSFDPV	10
	Human	NS5A	2255	LMVDSFDPV	9
	Human	NS5B	2505	LLDSHYESV	9
	Human	NS5B	2604	RLIVYPDLGV	10
	Human	NS5B	2734	SMGNTLTCYV	10
	Human	NS5B	2759	MLVCGDDLVV	10
	Human	NS5B	2869	MVLMTHFFSV	10
8	Human	NS5B	2870	VLMTHFFSV	9
	Human	NS5B	2870	VLMTHFFSVL	10
	Human	NS5B	291	RLHGLEAFSL	10
	Human	NS5B	2989	RLLDLSSWFT	10
	Human	NS5B	3016	RLLLLGLLLL	10
	Human	NS5B	3017	LLLLGLLLL	9
	Human	NS5B	3018	LLLGLLLLCV	10
	Human	NS5B	3019	LLGLLLLCV	9
	Human	NS5B	3021	GLLLLCVGV	9

Table S2E. **Overlapping peptides spanning the entire HCV genotype 3a potentially recognized by all HLA-restricted HCV-specific CD8^{+} T cells.**

Pool	AA	Protein	N. peptides	Average pep. lenght	Overlapping residues
**1**	1-191	Core	29	13-18	11-12
	192-383	E1	28	16-18	11-12
**2**	384-752	E2	57	15-19	11
**3**	753-815	p7	8	17-18	11
	816-1032	NS2	33	12-18	11-12
**4**	1033-1365	NS3	50	15-19	11-12
**5**	1355-1663	NS3	47	15-19	11-12
**6**	1664-1717	NS4A	7	14-18	11
	1718-1978	NS4B	39	15-19	11-12
**7**	1979-2306	NS5A	50	13-19	11-12
**8**	2296-2434	NS5A	22	13-19	11-12
	2431-2637	NS5B	30	14-19	11-12
**9**	2627-3021	NS5B	60	14-19	11-12
